# Integrin-Dependent Activation of the JNK Signaling Pathway by Mechanical Stress

**DOI:** 10.1371/journal.pone.0026182

**Published:** 2011-12-13

**Authors:** Andrea Maria Pereira, Cicerone Tudor, Johannes S. Kanger, Vinod Subramaniam, Enrique Martin-Blanco

**Affiliations:** 1 Instituto de Biología Molecular de Barcelona (CSIC), Parc Cientific de Barcelona, Barcelona, Spain; 2 Nanobiophysics, MESA+ Institute for Nanotechnology and MIRA Institute for Biomedical Technology and Technical Medicine, University of Twente, Enschede, The Netherlands; King's College London, United Kingdom

## Abstract

Mechanical force is known to modulate the activity of the Jun N-terminal kinase (JNK) signaling cascade. However, the effect of mechanical stresses on JNK signaling activation has previously only been analyzed by *in vitro* detection methods. It still remains unknown how living cells activate the JNK signaling cascade in response to mechanical stress and what its functions are in stretched cells.

We assessed in real-time the activity of the JNK pathway in *Drosophila* cells by Fluorescence Lifetime Imaging Microscopy (FLIM), using an intramolecular phosphorylation-dependent dJun-FRET (Fluorescence Resonance Energy Transfer) biosensor. We found that quantitative FRET-FLIM analysis and confocal microscopy revealed sustained dJun-FRET biosensor activation and stable morphology changes in response to mechanical stretch for *Drosophila* S2R+ cells. Further, these cells plated on different substrates showed distinct levels of JNK activity that associate with differences in cell morphology, integrin expression and focal adhesion organization.

These data imply that alterations in the cytoskeleton and matrix attachments may act as regulators of JNK signaling, and that JNK activity might feed back to modulate the cytoskeleton and cell adhesion. We found that this dynamic system is highly plastic; at rest, integrins at focal adhesions and talin are key factors suppressing JNK activity, while multidirectional static stretch leads to integrin-dependent, and probably talin-independent, Jun sensor activation. Further, our data suggest that JNK activity has to coordinate with other signaling elements for the regulation of the cytoskeleton and cell shape remodeling associated with stretch.

## Introduction

Cells, whether in isolation or in tissues, invariably face and respond to a wide variety of external stimuli. These environmental perturbations can be chemical or physical, and the responses can be physiological, such as cellular homeostatic activities or morphogenetic movements, or pathological, such as malignant transformation or inflammation. While the analysis of cellular responses to chemical signals has been studied in great detail, the elements involved in the recognition of physical inputs, e.g. hypoxia, osmotic shock, ionizing radiation or mechanical stretching, and the mechanisms transducing and implementing cell responses to these stimuli remain barely analyzed. These responses include a variety of conserved adaptive behaviors such as wound healing, cell migration, extravasation, secretion and necrotic or apoptotic death [1].

Mechanical stress is a prominent physical stimulus sensed by cells. At the cellular level, mechanical cues can modulate almost all aspects of cell behavior including growth, differentiation, migration, gene expression, protein synthesis and apoptosis [2], many of them of important clinical interest, e.g. cancer metastasis, stem cell proliferation and differentiation and wound healing. In developmental terms, mechanical stress influences a wide variety of morphogenetic processes like germ band extension in *Drosophila* [3] or gastrulation in *Xenopus* [4]. It also controls specific physiological processes such as sound sensation by cells of the inner ear or blood flow across the endothelium [2]. Indeed, in some cases, organs and tissues adapt their morphologies and functions in response to acute or chronic mechanical stress [5], e.g. pressure overload causes cardiovascular hypertrophy, and muscle disuse results in atrophy.

One widely studied family of mechanosensors is the cell surface integrin family [6]. Integrin-mediated cell adhesion and signaling are crucial events for numerous biological processes such as morphogenesis, the immune response, cell growth, and differentiation as well as for cell survival [7]. Integrins function as non-covalent heterodimeric transmembrane receptors that are organized in focal adhesions (FAs) and link the extracellular matrix (ECM) to the actin cytoskeleton; they do not directly interact with actin filaments. A number of actin-binding proteins, including talin, α-actinin and filamin, have been identified as intermediates. Among these, talin was the first intracellular ligand shown to interact directly with integrin ß-subunit cytoplasmic tails [8]. Integrins can mediate the sensing of mechanical properties of the ECM by changing their affinity, conformation, clustering and recruitment, and by transducing these signals to the activation of downstream signaling cascades. Further, the molecular architecture of FAs suggests that mechanical force is itself essential for focal adhesion formation and maintenance. The observed diagonal orientation of talin in FAs could arise from actomyosin pulling of the talin tails relative to the integrin-bound talin heads, with the resulting intramolecular tension straightening or stretching talin [9]. Thus, via stretch-induced recruitment, talin may effectively serve as a molecular ruler that specifies focal adhesion molecular architecture [10].

The c-Jun–N-terminal kinases (JNKs) are stress-activated protein kinases that belong to the superfamily of mitogen-activated protein kinases (MAPKs). They, in the framework of a three-tiered module of kinases, are regulated within eukaryotic cells by a process mediated by members of the Ras and Rho families of small GTPases [11] [12] in response to diverse extracellular stimuli [13] [14]. JNKs become activated after exposure to inflammatory cytokines as well as to diverse stress inputs including UV irradiation or heat shock. JNKs phosphorylate the DNA binding protein c-Jun and increase its transcriptional activity. c-Jun is a component of the AP-1 transcription complex, which is an important regulator controlling the expression of multiple target genes. Importantly, the functions of JNKs are context dependent and their activities can promote cell differentiation, apoptosis or survival (reviewed in [15]) or act either as tumor suppressors [16] or protumorigenic mediators [17] [18].

Mechanical force or mechanical stresses are known to modulate intracellular MAPK signaling cascades. The effect of mechanical stresses ranging from shear stress or fluid flow [19] to cell stretching [20] on MAPKs have been previously analyzed by *in vitro* detection methods such as western blot analysis using phosphospecific antibodies or by kinase assays after cell/tissue lysis. Cyclic stretch modulates the activities of p38 kinases, ERKs (Extracellular Regulated Kinases) and/or JNKs in many cell types, including mesangial cells [21], rat bladder smooth muscle cells [22], vascular smooth muscle cells [23], mouse fibroblastic L-929 cells [24] or human bronchial cells [25]. JNKs are also activated by static biaxial stretch in 3T3 cells [26].

*In vitro* analyses indicate that, in response to mechanical inputs, the kinetics of the activation/phosphorylation and dephosphorylation of MAPKs can be very diverse depending on the cell line and the parameters of the applied stress. Detailed dynamic analyses of the JNK signaling activity in response to stress in living cells, however, have been curtailed by the absence of appropriate tools and methodology. In this study, we used a robust and sensitive combination of FRET (Fluorescence Resonance Energy Transfer) and FLIM (Fluorescence Lifetime Imaging Microscopy) (see Text S1) with a dJun-FRET biosensor [27] to assess in real-time the activity of the JNK pathway in *Drosophila* S2R+ cells subjected to static mechanical stretch. We observed that cells subjected to static mechanical stretch revealed a significant increase in dJun-FRET biosensor phosphorylation, whose kinetics could be monitored live. Stretch also induced dramatic changes in cell morphology and actin and tubulin cytoskeleton dynamics. Further, we found that the basal activity of the dJun-FRET biosensor was extremely sensitive to the strength and type of cellular attachments. Remarkably, integrins, but probably not their attachment to the actin cytoskeleton via talin, were essential for stretch-mediated dJun sensor activation. We note however, that in the absence of either β-integrin (β subunit) or talin, cytoskeleton dynamics and cell shape were still affected by stretch. The potentially talin-independent JNK response to the mechanical stimulation of integrins at focal adhesions is a major element, but not the only one, in the regulation of the cytoskeleton and cell shape remodeling associated with mechanical stretch.

## Results

### FLIM measurements reveal the response to chemical activators and inhibitors of the JNK signaling cascade in living cells

We have previously engineered a dJun-FRET biosensor to conduct cell-based RNA interference (RNAi) screens by ratiometric fluorescence analysis to systematically investigate the JNK activity in various genetic backgrounds [27]. We have now used robust quantitative FLIM analysis to analyze specific cellular responses to mechanical stress. The lifetimes of the donor (mCFP) for a selection (∼75) of Regions of Interest (ROIs) comprising individual cells in a field of view were calculated from frequency-domain FLIM images (see Materials and Methods).

**Figure 1:**
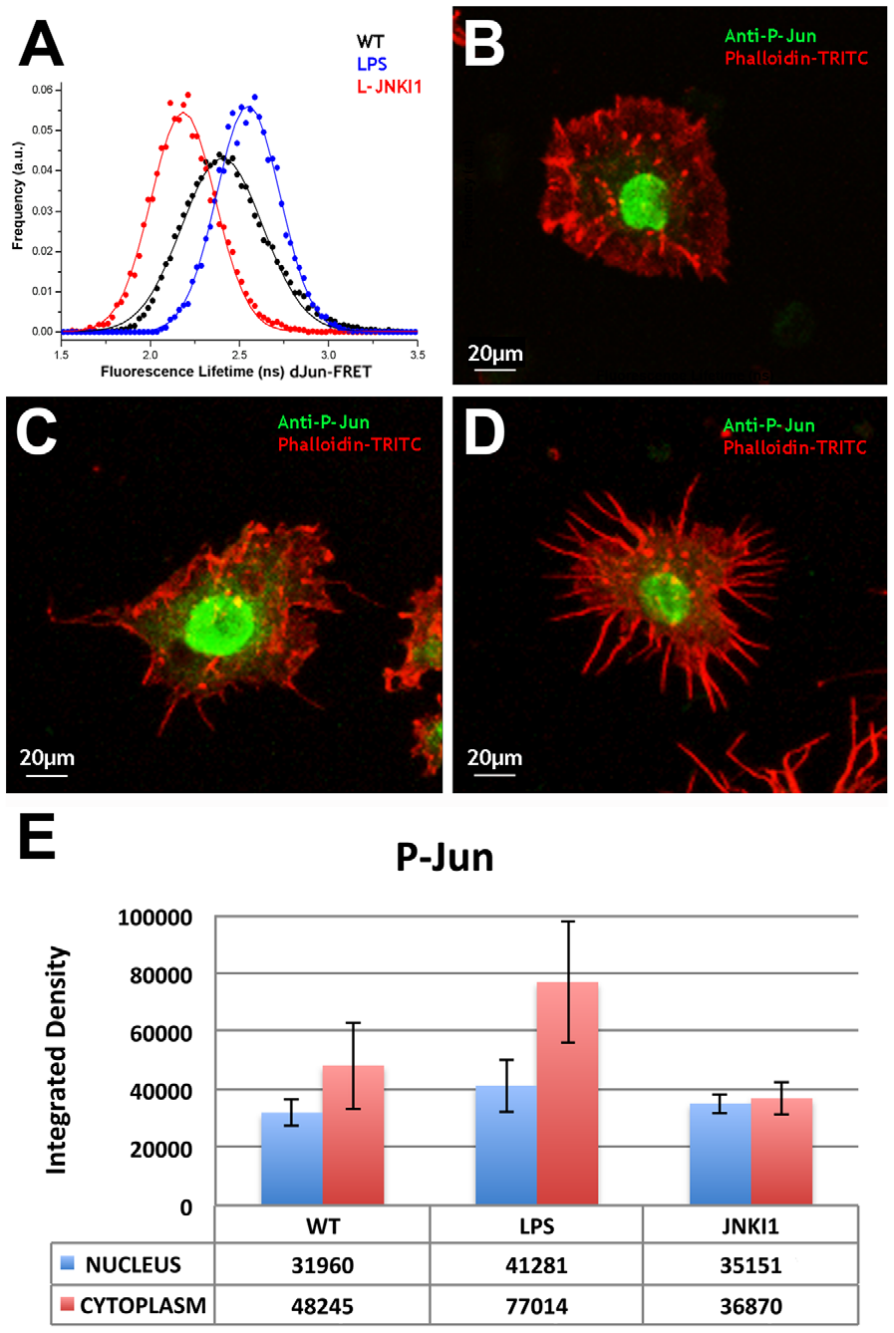
FRET-FLIM quantification of dJun-FRET biosensor is a readout of the activity of the JNK pathway in response to chemical agonists and antagonists. S2R+ cells were transiently transfected with dJun-FRET (A) biosensor, and fluorescence lifetimes (FL) of mCFP were collected 48 hours post transfection. Cells were left untreated (black) or subjected to treatment with LPS, a JNK signaling activator (red) or L-JNKI1, a JNK inhibitor (blue) for 2 hours before FLIM measurements. Curves represent FLIM data recorded from ∼75 cells for each condition. The chemical activator and inhibitor modulated the donor FL of dJun-FRET, while no effect was observed on the controls. A direct measurement of sensor activity on S2R+ cells plated on plastic was performed. Untreated cells (B) or those treated with LPS (C) or L-JNKI1 (D) were stained with anti-Phospho-c-Jun antibody and phalloidin-TRITC. P-Jun staining was quantified by calculation of the average integrated density (the product of Area and Mean Gray Value) of ∼100 cells (nucleus and cytoplasm) (E). LPS treatment led to morphological changes in S2R+ cells (from a pseudopolygonal flat shape to a filopodia-rich compacted aspect) and a statistically significant (p<5×10^−7^) increment of p-Jun staining (cytoplasm). L-JNKI1 treatment yielded cells with numerous multibranched thick filopodia and a statistically significant (p<5×10^−5^) decrease in P-Jun levels (cytoplasm).

S2R+ cells plated on plastic were transfected with either the dJun-FRET biosensor or mCFP and mCFP-dJun controls (see Materials and Methods), replated and cultured for 24 hours on collagen-coated silicone membranes in the absence of serum before any treatment. In resting, serum starved conditions the average fluorescence lifetime (FL) of the mCFP donor of the dJun-FRET biosensor in S2R+ cells was 2.40±0.22 ns. Activation of the pathway by treatment with 10 µg/ml Lipopolysaccharide (LPS), a known activator of the JNK pathway, for 2 hours, resulted in a reduction of the FL to 2.18±0.18 ns (Figure 1A and Figure S1). Considering the number of cells measured (n ∼75), these shifts in the FL distributions are statistically highly significant. S2R+ cells separately transfected with the control plasmids mCFP - lacking the Jun phosphorylation domain - and mCFP-dJun – lacking the YFP acceptor domain - (Figure S2) do not show any alterations to the average FL upon treatment with LPS; mCFP (2.65±0.14 ns before and 2.64±0.15 ns after LPS treatment) and mCFP-dJun (2.65±0.14 ns before and 2.65±0.13 ns after LPS treatment). Note that in resting conditions the donor FL of the dJun-FRET biosensor and of the control plasmids are different. In the case of the control plasmids there is no acceptor, and thus no possibility of FRET. The somewhat shorter donor lifetime of the dJun-FRET biosensor in the resting state most likely reflects proximity of the donor and acceptor moieties, which increases upon activation. A reverse effect on donor FL was observed upon treatment of dJun-FRET biosensor transfected S2R+ cells with a JNK inhibitor. Treatment of dJun-FRET biosensor transfected S2R+ cells with L-JNKI1, a cell-permeable inhibitor of JNK including the minimal 20 aminoacid inhibitory sequence of IB1, which is not fluorescent. 10 µM L-JNKI1 led to a robust increase of FL to 2.54±0.17 ns in 2 hours (Figure 1A and Figure S1).

A further, direct assessment of JNK activity was performed with anti-Phospho-c-Jun (Ser73) antibodies by immunofluorescence staining of S2R+ cells plated on plastic. P-Jun levels were quantified by calculation of the average signal integrated density of ∼100 cells (nuclei and cytoplasm). Immunostaining showed increased intensity upon treatment with LPS (Figure 1C) when compared to untreated S2R+ cells (Figure 1B). Treatment with the JNK inhibitor L-JNKI1 (Figure 1D) slightly reduced total p-Jun staining. Interestingly, most of the observed differences in the level of Jun phosphorylation are restricted to the cells cytoplasm (Figure 1E), in accord with recent reports suggesting that the nuclear import of Jun is independent of its phosphorylation [28]. To confirm biosensor specificity, we measured the FL of S2R+ cells transfected with dJun-FRET, activated with LPS and then treated with L-JNKI1. L-JNKI1 was epistatic and reverted the donor FL in activated cells to resting values (from 2.14±0.17 ns to 2.40±0.15 ns) (Figure S3). Activation or inhibition of the pathway also led to drastic cellular morphological changes of the cells (see Figures 1B, 1C and 1D and Figure S1). These observations will be discussed below.

Altogether these experiments provide compelling evidence that the observed decrease in FL upon treatment with LPS, attributed to a conformational change bringing together the donor (mCFP) and acceptor (YFP) domains of the biosensor, was induced by increased phosphorylation in the presence of the activator. The increase in donor FL in the presence of inhibitors would be, consequently, the result of the displacement of the equilibrium between phosphorylated and non-phosphorylated forms of the biosensor towards its inactive form. Thus, FLIM measurement of the dJun-FRET biosensor constitutes a robust method to evaluate the level of activity of the JNK pathway in living cultured cells.

### Specificity and dynamics of dJun-FRET biosensor activation

**Figure 2:**
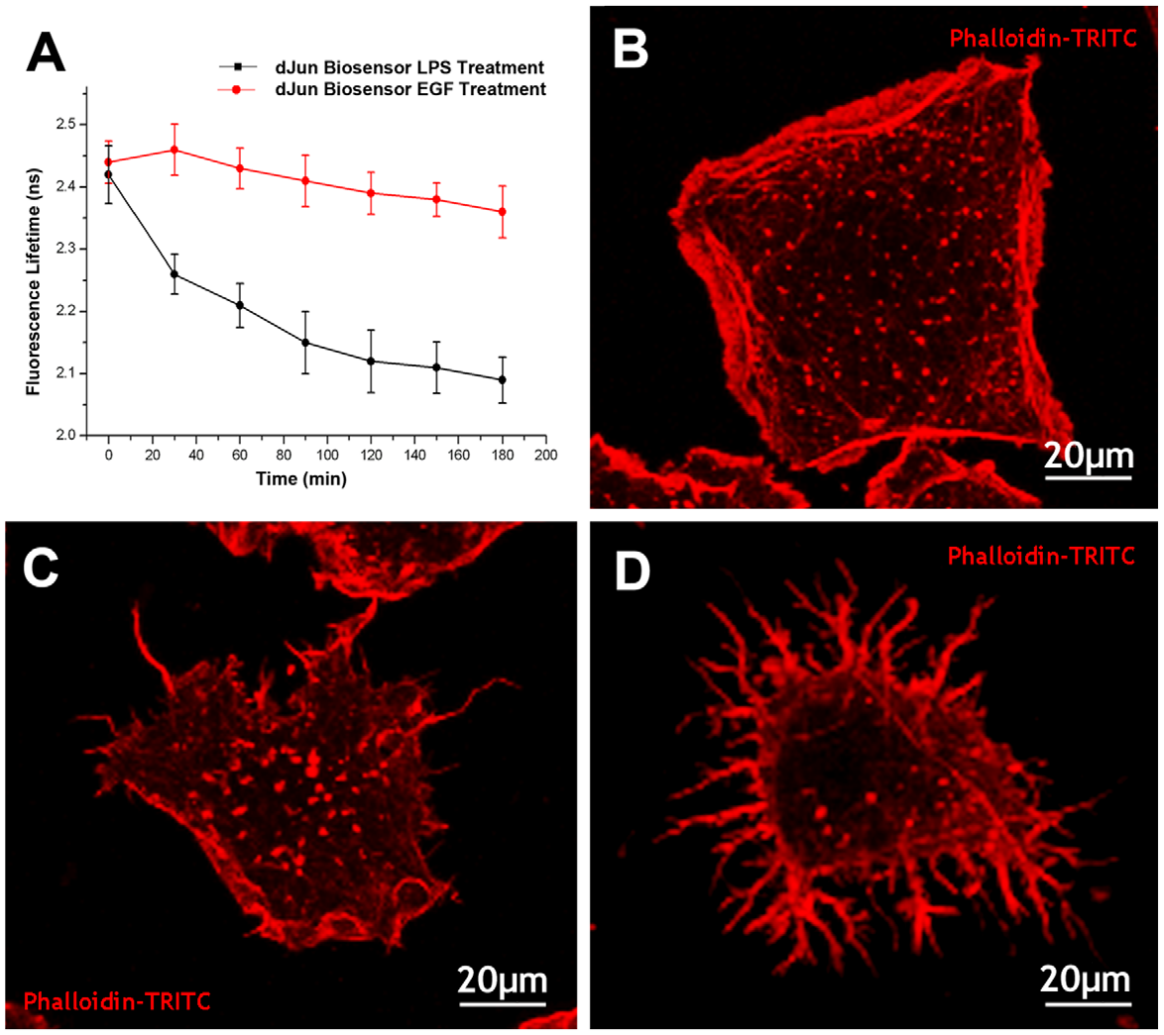
Specificity of the dJun-FRET biosensor. A) Time course of FL values for S2R+ cells transiently transfected with dJun-FRET biosensor and subjected to treatments with LPS (black) or Epidermal Growth Factor (EGF) (red). FLs of the donor (mCFP) were collected for 3 hours at 30 minutes intervals. In the presence of LPS, donor FL significantly decreases within 30 minutes, however, no significant shift was observed for 3 hours in the presence of EGF. B-D) S2R+ cells, serum starved for 24 hours, were plated on plastic, treated with the corresponding ligand for 2 hours, fixed and stained with phalloidin-TRITC. Untreated cells (B) show pseudopolygonal shape with peripheral accumulation of fibers and cytoplasmic spots of actin. LPS treated cells (C) present a compacted morphology and occasional thin filopodia, while EGF treated cells (D) display many highly branched short filamentous actin-rich protrusions.

To assess the dynamics of dJun-FRET biosensor activation by LPS, we collected FL for individual S2R+ cells plated on plastic at 30-minute intervals (Figure 2A). Average FL values as a function of time remained constant (2.44±0.03 ns), and correlated very well with those observed for cells plated on collagen-coated membranes. Upon addition of 10 µg/ml LPS a rapid decrease in FL values was observed. Steady-state activation of the dJun-FRET biosensor was reached in approximately 90 minutes, and remained fairly stable for at least 3 h. Equivalent treatment of plastic-plated S2R+ cells with EGF resulted in no significant changes in FLIM values for the dJun-FRET biosensor in this period of time. Different concentrations of EGF ranging from 50 ng/ml to 100 ng/ml were tested, all being neutral to JNK activity. Altogether, these data demonstrate the specificity of the biosensor. Remarkably, as noted above, the morphology of S2R+ cells changed under different treatments, LPS or EGF, when plated on plastic (Figure S1 and compare Figure 2B to 2C and 2D).

### Mechanical stress affects cell morphology and JNK activity

**Figure 3:**
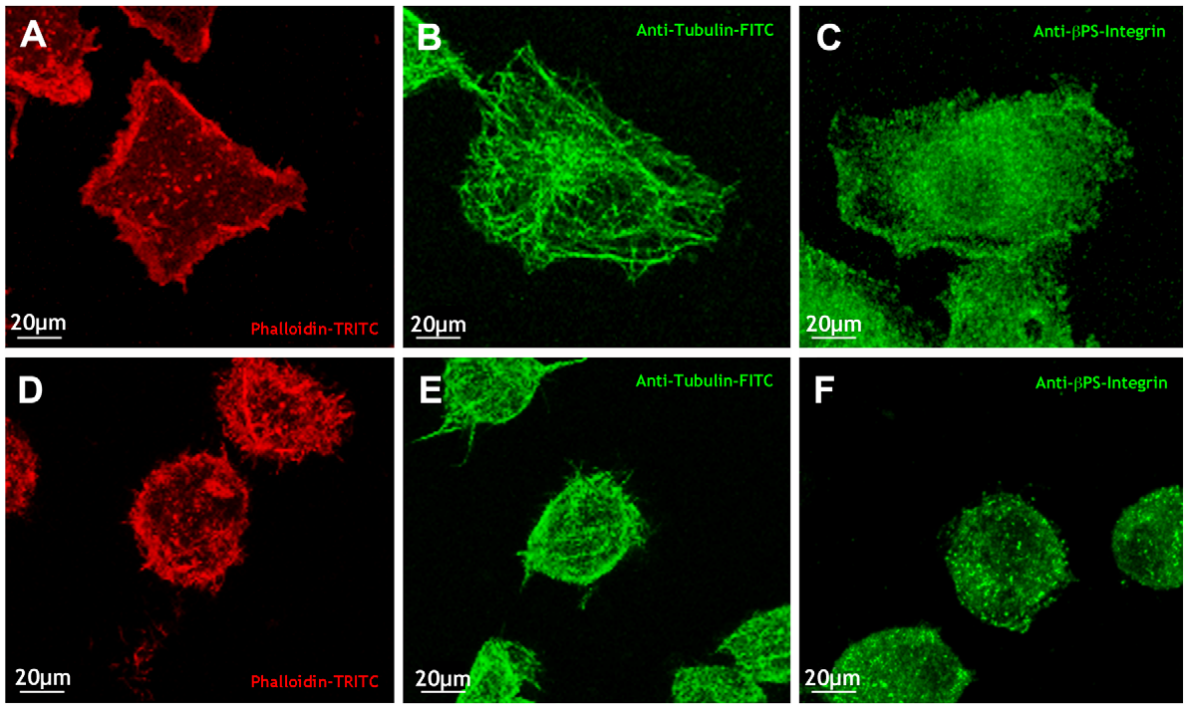
S2R+ cells respond to mechanical stress by changing their morphology and altering their cytoskeletal network. S2R+ cells plated on collagen-coated silicone membranes showed a polygonal shape with actin rich stress fibers and lamellipodia at their periphery (A) and distinctly arranged long microtubules spreading out and intermingling in the center (B). Anti- β-integrin antibodies show high expression at the periphery and dispersed cytoplasmic distribution (C). Upon subjecting cells to static stretch for 1 hour, S2R+ cells rounded up showing spotty cytoplasmic polymerized actin (D), short and diffuse microtubules (E) and punctate expression of β-integrin (F). Red - Phalloidin-TRITC; green - anti-Tubulin-FITC and anti- β-integrin antibodies.

To analyze the effect of cell stretching, S2R+ cells were plated on collagen-coated silicone membranes (on which S2R+ cells exhibit an intermediate level of sensor activation in resting conditions), allowed to adhere overnight at 25°C in the absence of serum, and then exposed to 2.5% static stretch on a Stage Flexer vacuum device. The morphologies of unstretched and stretched cells were evaluated in fixed preparations stained with anti-Tubulin FITC conjugated antibody and Phalloidin-TRITC. Unstretched cells showed a flat expanded shape displaying thick actin fibers and lamellipodia at the periphery, distinctly arranged microtubules which spread out at the periphery and intermingle at the center where the cell domes up, and a diffuse expression of β-integrin that accumulates at the periphery (Figures 3A to 3C). Stretched cells underwent a clear change in shape, rounding up and showing spotty actin and β-integrin expression and diffuse cytoplasmic tubulin staining (Figures 3D to 3F). Occasionally, stretched cells displayed small, loose filopodia-like protrusions. Morphological parameters are quantified and presented in Figure S4 (WT cells under unstretched and stretched conditions).

To evaluate the kinetics of the response to stretch of cells transfected with pMT-tubulin-GFP, we plated them on collagen-coated silicone membranes and imaged them at 3-minute intervals in unstretched/resting conditions and after induction of static stretch. Resting S2R+ cells showed a very stable spread out morphology and did not exhibit any spontaneous rounding up in our experimental timeframe (up to 4 hours live imaging). In response to static stretch we observed a fast increase in cytoskeletal dynamics (within minutes), concomitant with a drastic morphological change of the cells, which rounded up, collapsing their cytoplasms towards the nuclei (Movie S1). A full morphological transition was reached in around 60 minutes, and remained at a steady state for a few hours. We further observed that cells subjected to stretch for more than 12 hours did not detach from the substrate.

**Figure 4:**
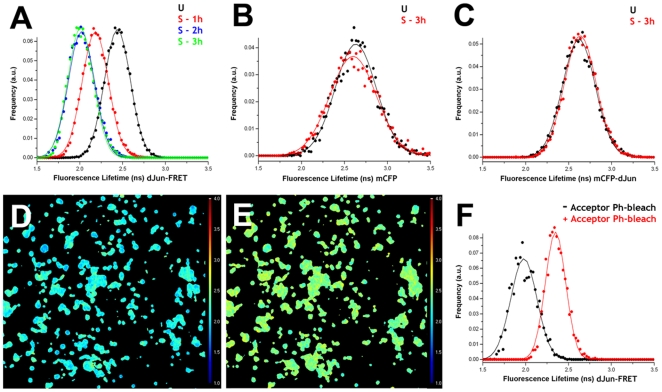
Mechanical stress activates the JNK pathway. S2R+ cells transiently transfected with dJun-FRET biosensor (A) or the controls mCFP alone (B) and mCFP-dJun (C) were plated on collagen-coated silicone membranes on a Stage Flexer set up. Donor mCFP FL was collected before stretching (black) and at different times after continuous static stretch (1 hour-red in A, 2 hours-blue in A, and 3 hours-green in A and red in B and C). dJun-FRET sensor activity increased upon stretching, reaching a maximum after 2 hours. FL of controls was not affected. Donor mCFP FL from a specific region of interest containing ∼15–30 cells were collected after 3 hours of mechanical stretch (D). Thereafter, the acceptor mYFP was photobleached (95%) and donor mCFP FLs were re-collected (E). Panel F shows the donor mCFP FL histograms for the dJun-FRET biosensor before (black) and after (red) acceptor (mYFP) photobleaching. Altogether, these data showed that FL changes observed after mechanical stretch can be attributed directly to energy transfer between the donor and acceptor fluorophores in the dJun-FRET biosensor.

To check whether the activity of the JNK pathway is modified in response to mechanical stretch, S2R+ cells transfected with the dJun-FRET biosensor were plated on collagen-coated silicone membranes attached to a Stage Flexer device. As described above, cells were allowed to adhere to the substrate and eventually subjected to mechanical stretch. The average FL for the unstretched cells (2.43±0.15 ns) matched previous data obtained on collagen-coated silicone membranes cultured in standard chambers. The FL of cells subjected to static stretch was significantly reduced after 1 hour (2.18±0.15 ns) and reached a plateau after 2 hours of continued exposure (2.01±0.15 ns), when the dJun-FRET biosensor reached phosphorylation saturation (Figure 4A). A detailed time course analysis showed that a substantial decrease in FL could be observed within 20 minutes upon stretching (Figure S5). As control experiments, S2R+ cells transfected with mCFP or mCFP-dJun were exposed to the same mechanical manipulations. No significant shifts in FL values were observed in these cases. For mCFP alone an average value of 2.63±0.24 ns was obtained in the absence of stretch and 2.60±0.27 ns after 3 hours of mechanical stress (Figure 4B). In the case of mCFP-dJun, an average value of 2.62±0.19 ns was obtained in the absence of stretch and 2.63±0.18 ns after 3 hours of static stretch (Figure 4C).

Finally, an acceptor (mYFP) photobleaching experiment was performed as a control. S2R+ cells expressing the dJun-FRET biosensor were subjected to continuous mechanical stretch for 3 hours. A specific region of interest (ROI) containing ∼15–30 cells was selected and the FLs for mCFP were collected (Figure 4D). An average FL of 1.97±0.15 ns was calculated (Figure 4F). After image acquisition, the acceptor mYFP was photobleached (∼95%) in the same ROI and the FL for mCFP collected again (Figure 4E). If the reduced FL of mCFP after 3 hours of static stretch is due to energy transfer between the mCFP and mYFP fluorophores of the biosensor, then by photobleaching the acceptor mYFP the energy transfer should be abolished and a recovery of mCFP FL should be observed. Indeed, the average FL obtained for mCFP after mYFP photobleaching was 2.34±0.12 ns (Figure 4F), significantly higher than that observed before mYFP photobleaching. Altogether, these results suggested that the JNK pathway is activated as a consequence of the applied mechanical stretch stress. This activation temporally correlated with the kinetics of the morphological changes observed upon cell stretching.

### Attachment dependent activation of the JNK pathway

**Figure 5:**
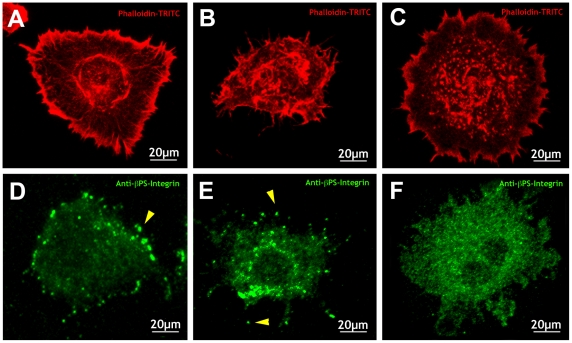
S2R+ cells exhibit different morphologies and matrix attachments on different substrates. S2R+ cells were plated on glass (A, D), collagen-coated glass (B, E) and Con-A-coated glass (C, F). They were stained with phalloidin-TRITC (A–C) and anti- β-integrin antibodies (D–F) and visualized by fluorescence confocal microscopy. S2R+ cells plated on glass shrank around their nuclei showing extensive actin-rich lamellipodia and occasional short filopodia and punctate β-integrin expression at the margins (arrowhead) and dispersed all over the cytoplasm and the cell periphery. Cells plated on collagen-coated glass show abundant long and thin protrusions with punctate β-integrin at their tips (arrowheads). Cells plated on Con-A-coated glass show spread out morphologies, dense peripheral actin staining and circular bundles of actin around the nucleus. These cells present diffuse, just above background, β-integrin staining.

Survival and growth of cells in culture are strongly influenced by the material properties of the culture substrate, and by its coating with attachment factors. Cell morphology is also determined by the surface to which the cell is attached, as cell shape is modulated by the surface-dependent rearrangements of the fibrous elements of the cytoskeleton, microtubules, and microfilaments. We found that the shapes of S2R+ cells are extremely sensitive to the characteristics of the substrate on which they are grown. As described above, cells plated on collagen-coated silicone membranes (Figure 1B) or on plastic (Figure 2B) showed a related pseudo-polygonal stretched shape with accumulation of actin stress-fibers at the periphery and actin-rich spots randomly distributed in the cytoplasm. The morphologies of S2R+ cells dramatically changed when plated on uncoated or on collagen- or Concanavalin-A (Con-A)-coated glass. On uncoated rigid glass, cells are flat and contract their bodies around the nuclei showing extensive actin-rich lamellipodia and multiple short spikes (Figure 5A). When glass was coated with collagen, seeded S2R+ cells showed a contracted shape and disordered conspicuous actin-rich long protrusions (Figure 5B). Remarkably, most S2R+ cells plated on Con-A-coated glass exhibited a drastically different very flat shape with a highly developed, radially symmetrical actin cytoskeleton consisting of a dense peripheral network at the extreme periphery of the cells, a second central zone of lower actin density, and a third circular bundle of filaments that surrounded the nucleus (Figure 5C).

Besides morphological differences, S2R+ cells plated on different substrates present distinct distributions of focal adhesions as monitored by expression of ß-integrin (Myospheroid - Mys). On uncoated glass, Mys expression is dispersed all over the cytoplasm, the periphery, and in punctae in short protrusions (Figure 5D), while on collagen-coated glass, cells showed an abundant spotty Mys expression mostly located surrounding the nuclei and at the periphery in punctate spots at the tips of long actin-rich protrusions (Figure 5E). Distinctively, cells plated on Con-A-coated glass only showed diffuse Mys staining slightly above the background, suggestive of the absence of focal adhesions (Figure 5F).

**Figure 6:**
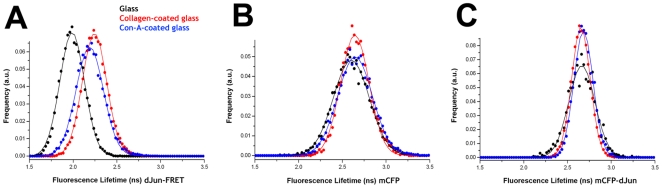
Attachment dependent activation of the JNK pathway. S2R+ cells transiently transfected with dJun-FRET biosensor (A) or the controls mCFP alone (B) and mCFP-dJun (C) were plated on different surfaces and the mCFP donor FLs were collected 48 hours post transfection. dJun-FRET biosensor activity (A) was highest on cells plated on glass (black), and much less pronounced on collagen-coated glass (red), and on Con-A-coated glass (blue). FL of control biosensors was not affected by the substrate (panels B, C).

To explore if the attachment to the different substrates could affect the activity of the JNK pathway in S2R+ cells, we plated them on different surfaces and used FLIM to measure the average FL values of the dJun-FRET biosensor as described above. Concomitant with the morphological changes, we observed that the FL values of the dJun-FRET biosensor (and hence JNK activity) are strongly altered by the choice of attachment surface. S2R+ cells attached to uncoated rigid glass surfaces resulted in a high level of sensor activation (FL value of 1.98±0.14 ns), while cells seeded on collagen or Con-A-coated glass surfaces exhibit intermediate levels of activity (2.24±0.14 ns and 2.19±0.15 ns respectively) (Figure 6A). One technical reason for these differences in the observed donor FL could be the different refractive indices of the surfaces used. FLs of fluorophores are known to be influenced by the refractive index of the surrounding environment [29]. Control experiments were thus performed to check whether refractive index differences could lead to the observed fluctuations of dJun-FRET biosensor FL. S2R+ cells expressing mCFP-dJun and mCFP control constructs were plated on different surfaces (plastic, glass, collagen on glass or Con-A on glass) and FLs were measured by FLIM. These controls showed that FL values for mCFP-dJun and mCFP exhibit no significant differences on the different surfaces. The average FL values for mCFP were 2.60±0.20 ns for glass, 2.64±0.16 for collagen, and 2.64±0.20 ns for Con-A (Figure 6B), while values for mCFP-dJun were 2.66±0.15 for glass, 2.65±0.15 ns for collagen and 2.68±0.15 for Con-A (Figure 6C). From these data we concluded that the differences in FL for the dJun-FRET biosensor are not influenced by the refractive index of the surface.

In summary, the level of JNK activity and the morphologies of S2R+ cells are affected by the mechanisms employed to attach to the substrate matrix (Figure S6). These changes most probably depend on differences in the adhesion components linking the cell interface to the supporting surface and on the signaling events elicited as a result of such attachment.

### JNK signaling activation by mechanical stress depends on integrins

**Figure 7:**
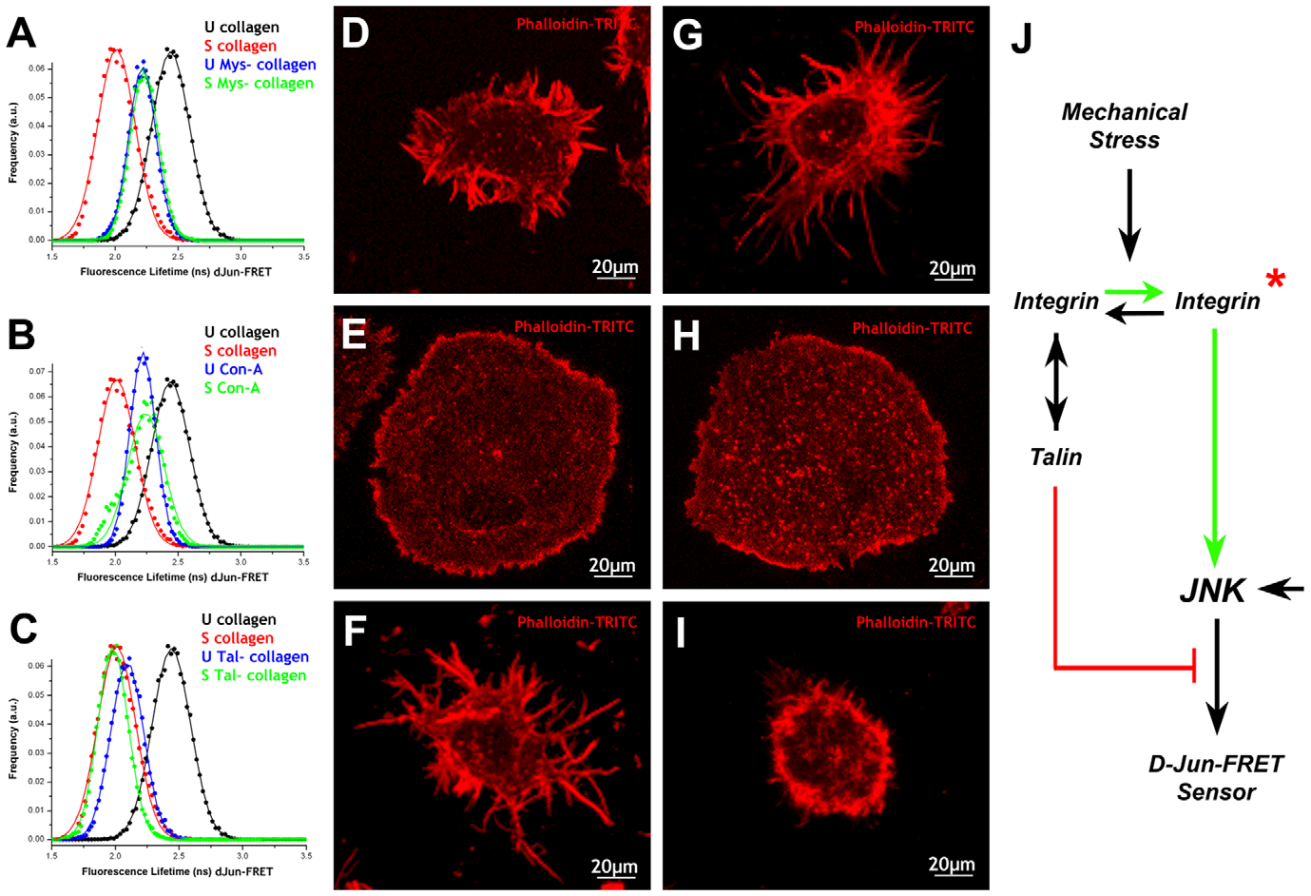
Integrins and talin modulate the JNK response to mechanical stress. S2R+ cells transiently transfected with dJun-FRET biosensors were plated on collagen- (A – treated with Mys dsRNA and C – treated with talin dsRNA) or Con-A-coated (B) silicone membranes on a Stage Flexer set up. Donor mCFP FLs were collected before stretching (blue) and at 3 hours after continuous static stretch (green). In each graph are also represented the data obtained for untreated cells plated on collagen-coated silicone membranes (black– before stretch; red– 3 hours after continuous stretch). In the absence of Mys, talin or by plating cells on Con-A, the activity of JNK increases in relation to WT cells at rest. However, only the talin minus cells were able to increase their dJun-FRET sensor activity upon stretch. Mys minus cells plated on collagen-coated silicone membranes show a pseudopolygonal shape and emit multiple thick short protrusions (D). They rounded up upon stretch emitting very thin filopodia (G). Talin minus cells were more rounded and showed few, long branched filopodia (F). They collapsed in response to stretch showing occasional, very short, thin protrusions (I). Cells plated on Con-A-coated silicone membranes showed a flat shape (E) that was not affected by mechanical stress (H). The data acquired suggests a hierarchical model for the roles of β-integrin and talin regulating the level of Jun sensor activation of S2R+ cells and its response to mechanical stress (J). In the absence of mechanical input both β-integrin and talin restrain the activity of the JNK signaling. In the absence of any of them, the pathway gets moderately activated in response to an independent input. Mechanical stress results in β-integrin activation and the establishment of a, probably talin-independent, positive contribution to JNK signaling leading to a maximum level of dJun-FRET biosensor activity.

To analyze the role that matrix attachment and integrins could have in the dJun-FRET biosensor response to mechanical stress, we interfered with Mys expression by RNAi in S2R+ cells (see [30]). Inhibition of Mys in unstretched cells resulted in a reduction of the dJun-FRET biosensor FL from 2.43±0.15 ns in untreated S2R+ cells to 2.21±0.11 ns, suggesting that integrins somehow inhibit the dJun-FRET biosensor activation at resting conditions. Notably, when these cells were subjected to static stretch, no activation of the pathway was observed. Upon stretching Mys-RNAi expressing cells the dJun-FRET biosensor FL remained unchanged at 2.23±0.11 ns in contrast to a reduction in FL to 2.01±0.15 ns in WT cells (Figure 7A). Thus, in the absence of Mys, mechanical stress fails to induce sensor activation. Further, S2R+ cells plated on Con-A, which showed reduced FL (2.21±0.11 ns) in resting conditions (in the range of Mys RNAi treated cells), also did not exhibit any change in the activity of the JNK pathway (FL 2.24±0.15 ns) in response to mechanical stress (Figure 7B). On the contrary, interfering with the expression of talin (see [30]), an adaptor of integrins to the actin cytoskeleton [10], which itself resulted in a reduction of the dJun-FRET biosensor FL to 2.10±0.13 ns at rest, did not prevent the further activation of the sensor by mechanical stress (FL 1.98±0.12 ns) to the level of WT cells (2.01±0.15 ns) (Figure 7C). The difference of the dJun-FRET biosensor FL between unstretched and stretched talin minus cells is statistically significative (p<0.001).

Mys and talin RNA interference resulted in remarkable reorganizations of shape when compared to the WT condition. In both cases, S2R+ cells shrink their cell bodies, but while Mys minus cells keep a pseudopolygonal shape and emit multiple actin-rich thick short protrusions (Figure 7D), talin minus cells are more rounded and show few, long and occasionally branched filopodia (Figure 7F). In contrast, cells plated on Con-A-coated silicone membranes showed the flat extended shape already observed on cells plated on Con-A-coated glass (Figure 7E). Mechanical stretch, as in the WT cells, resulted in shape alterations in Mys and talin RNAi treated cells. Despite no change in the level of dJun-FRET biosensor activation, Mys minus cells further round up upon stretch and emit radially arranged very thin filopodia (Figure 7G). Talin minus cells collapse in response to stretch (Figure 7I), only showing occasional very short, thin protrusions (a phenotype resembling that of untreated cells under mechanical stress). The flattened shape of S2R+ cells plated on Con-A was not further affected (Figure 7H). These morphological data are summarized in Figure S4.

## Discussion

### FRET/FLIM is a robust method for quantification of JNK signaling activity

We have previously analyzed the activity of the JNK signaling pathway in *Drosophila* BGL-2 using a dJun-FRET biosensor [27]. These analyses allowed the identification of diverse regulators and signaling networks modulating the activity of *Drosophila* JNK. An essentially identical Jun-FRET biosensor (JNKAR1) has been recently developed and applied to quantitative single-cell analysis of JNK activity in HeLa cells [31]. In these studies ratiometric donor/acceptor measurements were applied to evaluate the degree of energy transfer.

Although ratiometric approaches to FRET quantification are well established, they suffer from the major drawback of spectral bleed-through or cross talk. FLIM serves as a robust alternative method to measure energy transfer [32], and is not subject to spectral cross-talk. A primary advantage of FLIM is that lifetime measurements are independent of changes in excitation intensity or fluorophore concentration. When acceptor molecules are in the close vicinity (1–10 nm) of donor fluorophores, energy transfer leads to a decrease in the donor FL. Thus, one can quantitatively measure the existence and extent of energy transfer by simply monitoring the FL of the donor fluorophore. FLIM has recently been used to study interactions of growth factor receptors, to elucidate the presence of micro clusters in immunological synapses, to study integrin-effector binding, to monitor intracellular ion concentrations during neural development, to monitor synaptic interactions, to study the structure and function of endosomes and for in situ analysis of tyrosine phosphorylation networks on cell arrays amongst other applications [33] [34] [35].

We have used a frequency-domain FLIM method, in which the excitation is sinusoidally modulated, to measure FLs of the donor. Using this method, we have found that the dJun-FRET biosensor responds to specific JNK signaling activators and inhibitors in a dose- and time-dependent manner. Although the dynamic range of the sensor is small, it is sensitive enough to identify minute changes of activity in a statistically relevant way. Furthermore, we were able to follow dynamically in living cells the modulation of the JNK pathway, opening the way to live analysis and high-throughput assays by automated FLIM (see [35]).

### Modulation of dJun-FRET biosensor activation by attachment to the substrate

We found that the choice of underlying substrate modulates the shape, cytoskeletal distribution, and attachments of S2R+ cells. Cells directly attached to glass are flat, polygonal, and exhibit multiple focal adhesions and a peripheral localization of ß-integrin receptors, while those plated on collagen (coated on silicone membranes or glass) show rounded shapes and ß-integrin expression around the nuclei and at specific punctae at the tips of filopodia. Remarkably, changes in the levels of sensor activation in S2R+ cells are associated with specific cell shapes and the number and density of focal adhesions. Thus, S2R+ cells plated on glass exhibit low FL values (high level of activation), while cells plated on collagen present higher FL values (more evident on silicone membranes). A different pattern was observed for cells plated on Con-A, which spread isotropically and present just diffuse ß-integrin expression. S2R+ cells do not build focal adhesions on Con-A, which interacts with glycosyl residues in terminal positions of ramified structures from ß-Glycans [36]. These cells show intermediate levels of dJun-FRET biosensor activity.

### Cell stretching modulates dJun-FRET biosensor activation

A transient activation of JNK signaling in response to mechanical stretch has been reported for many different cell types in culture. This ranges from 2 minutes in glomerular mesangial cells [21] to 30–60 minutes in bladder [22] and vascular smooth muscle cells [22], skeletal muscle microvascular endothelial cells [37], and renal epithelial cells [21]. In these cases, experimental data support, and kinematic models predict, that, after an initial burst of the rate of stress fiber turnover in response to experimental uniaxial stretch, a rapid decrease follows as stress fibers align away from the direction of stretch. These dynamic cytoskeletal changes would dynamically modulate the time course of JNK activation [38]. On the other hand, robust sustained JNK activation has been observed when cells (bovine aortic endothelial cells or vascular smooth muscle cells) are subjected to cyclic equibiaxial stretch [39] [40]. Cells exposed to this non-polarized stretch regime are unable to reorient their stress fibers in a direction that avoids perturbations in stretch, keeping elevated their rate of stress fiber turnover and as a result their JNK activity [38].

In this study, S2R+ cells subjected to 2.5% static mechanical stretch rapidly rearrange their actin fibers, microtubular network and matrix attachments within 60 minutes of induction. They round up to adapt to the perturbations of the external environment and stay stable for at least 3 hours. Likewise, just after stimulus, the JNK pathway increases its activity very rapidly reaching a plateau in less than 2 hours, remaining steady for the whole period of analysis. In *Drosophila* S2R+ cells, the sustained activation of the JNK cascade caused by multidirectional static stretch might be a reflection of the steady active remodeling of their cytoskeleton as a consequence of the lack of directionality of the matrix strain.

### How does JNK signaling get activated in response to mechanical stress?

As discussed above, S2R+ cells when plated on collagen alter their ECM attachments and cytoskeleton in response to mechanical stretch. Whether the activation of the JNK pathway is the cause or the effect of this remodeling remains to be addressed. It has been postulated that transduction of matrix forces into intracellular signals occurs through force-dependent conformational changes in proteins connected to the cytoskeleton [41] and steady high rate of cytoskeleton/attachments turnover and anisotropic tension can lead to sustained JNK activation [38]. Accordingly, stretch-induced upregulation of JNK activity may occur as a result of the tensile properties of actin fibers and their associated integrin-matrix bonds. Indeed, it has been observed that JNK signaling can be stimulated as a result of integrin activation in response to mechanical strain [26]. However, we have found in S2R+ cells that although integrins are essential for dJun-FRET biosensor activation in response to mechanical stress, their attachments to actin via talin may not be. In the absence of talin, the sensor is fully activated by mechanical stretch and cells round up and remodel their cytoskeleton in a manner similar to WT cells. On the contrary, in resting conditions, the presence in WT cells of Mys and talin resulted in low sensor activity (Figure 7J). In their absence, or in cells plated on Con-A with no functional focal adhesions, the dJun-FRET biosensor, in response to an independent and as yet not identified input, reaches a moderate/intermediate level of activation at rest. We hypothesized that mechanical stretch leads to the activation of integrins at focal adhesions on collagen-plated cells and promotes a, probably talin-independent, full activation of the JNK pathway.

### What is the function of the JNK signaling in stretched cells?

In nearly all model cellular systems the most frequently observed consequence of mechanical stretch is apoptosis. In myoblast C2C12 cells or porcine retinal pericytes, stretch induces the production of abundant reactive oxygen species (ROS) controlling caspase-3 activation [42] [43]. Further, in mesenchymal stem cells, tensile strain leads to activation of Stress Activated Channels (SACs) and cell death [44]. In all these cases the JNK pathway acts as a necessary intermediate signaling element and the inhibition of JNK activity by RNAi or specific inhibitors blocks caspase 3 activation increasing cell survival. A second major effect of JNK activation in response to mechanical stretch is the production of cytokines and metalloproteinases. Thus, interleukin IL-8 is upregulated in response to JNK signaling after positive pressure ventilation of mice *in vivo* or cyclic stretch of A549 cells, a human alveolar epithelial cell line [45]. In addition, static mechanical stretch induces matrix metalloproteinases 2 and 14 (MMP2 and MMP14) expression in microvascular and human umbilical vein endothelial cells [37] [46]. In these models, inhibition of JNK suppressed stretch-induced MMP2 and MMP14 protein and mRNA.

The response of S2R+ cells to mechanical stretch, however, does not seem to lead to cell death or increased proliferation (data not shown). Instead it appears to be linked to active cytoskeleton and matrix attachment rearrangements and we have found that it depends on the expression of ß-integrins. In this sense, the process looks more related to the effect of stretch on alveolar epithelial cells that results in increases in paracellular permeability, which is also associated with a rise in JNK activity [47]. JNK activation in these cells is linked to phosphorylation of tight junction components such as occludin and ZO-1 and a drop in occludin expression. Increased phosphorylation of these proteins triggers the reorganization of the tight junction complex, promoting junction disassembly and increasing epithelial permeability.

S2R+ cells are extremely plastic and their form can be altered by a wide variety of chemical and physical insults. The absence of Mys or talin, or the plating of cells on Con-A, resulted in profound alterations of cell shape and rearrangement of the actin cytoskeleton differing from the WT. These observations imply that focal adhesions and the link between integrin and the actin cytoskeleton via talin are key factors in modulating their morphology. In response to mechanical stress, a full stable rearrangement of cell shape correlates with maximum activation of the dJun-FRET biosensor in WT or talin minus cells. However, intermediate levels of sensor activity as those observed in Mys minus or Con-A plated cells under stretch do not necessarily result in equivalent morphologies. Thus, the remodeling of cell shape and the dynamical rearrangement of the cytoskeleton of S2R+ cells in response to stress is not just the outcome of the attained level of sensor activation.

Our results showing sustained JNK signaling activation and stable morphological and behavioral changes in response to stress support the hypothesis that alterations in the cytoskeleton and matrix attachments act as regulators of JNK signaling in response to mechanical stretch. Otherwise, the JNK pathway might effectively feedback to modulate cell adhesion and cytoskeleton dynamics. RNAi interference in elements of the JNK cascade will potentially shed light onto this puzzle.

## Materials and Methods

### Materials

Lipopolysaccharide (LPS), JNK inhibitor SP600125, epidermal growth factor (EGF), mouse anti β-Tubulin-FITC, Goat anti-mouse Alexa488 (1∶200) and Phalloidin-TRITC were purchased from Sigma-Aldrich. Mouse anti- β-integrin (1∶10; CF.6G11 was obtained from the Developmental Studies Hybridoma Bank (DSHB). The JNK Inhibitor 1 (L-JNKI1) was purchased from Alexis Biochemicals. The 43 mm silicone rubber membranes coated with collagen were from Flexcell International Corporation.

### Cell culture and transfection

*Drosophila* S2R+ cells [48] were grown routinely in Schneider's *Drosophila* medium (GIBCO, Invitrogen) supplemented with 10% heat inactivated Fetal Bovine Serum (GIBCO, Invitrogen) at 25°C. Penicillin and streptomycin were included at 100 units/ml and 100 µg/ml, respectively. Cells were seeded one day prior to transfection in 24-well tissue culture plates (Greiner Bio-One). Transfection of *Drosophila* S2R+ cells was performed with 2 µg/µl of plasmid at ∼80% confluency using Effectene reagent (Qiagen) following the manufacturer's instructions. The ligands LPS (10 µg/ml), SP600125 (100 ng/ml), L-JNKI1 (10 µM) or EGF (50 ng/ml) were added 2 h before starting FLIM measurements.

### Expression Constructs

FLIM analysis was performed in S2R+ cells transfected with *actin*-mCFP (mCFP), *actin*- mCFP-dJun (mCFP-dJun) or *actin*-dJun-FRET (dJun-FRET biosensor) reporters. The dJun-FRET biosensor for detecting JNK-mediated phosphorylation has been already described [27]. It is composed of two fluorophores, a monomeric CFP (mCFP) and a monomeric YFP (mYFP) flanking a JNK substrate sequence (modified Jun phosphorylation site) tethered by a flexible linker to a FHA2 phosphothreonine binding domain from the yeast checkpoint protein Rad53p. Phosphorylation of the substrate sequence triggers an intramolecular clamp with the FHA2 module causing a conformational change that brings the mCFP and mYFP moieties in close proximity and alters the amount of FRET from mCFP to mYFP. The *actin*-mCFP (mCFP) control reporter consists of the mCFP fluorophore cloned into the pAct5C expression vector. This reporter lacks the dJun and mYFP domains. The mCFP-dJun reporter is a plasmid containing the same sequence as the dJun-FRET biosensor except that the mYFP domain has been removed. The mCFP-dJun was built by PCR amplification of the FHA2-dJun-mCFP domains (1200 bp) with the following primers: 5′GTC ATA CTC GAG ATA ATG CCG TCG ACT TTT TAA CTT 3′ and 5′GAC TAT TCT AGA GCG GCC CAG CTC GTC CAT GCC GAG 3′ and subsequent subcloning in pAct5C.

### Immunostaining and imaging of cells

Cells were fixed with 4% PF for 20 minutes without shaking, rinsed with 1× PBS 2×15 minutes, then with 0.3% PBTriton-X100 3×15 minutes, incubated with phalloidin-TRITC/anti-tubulin FITC for 2 hours at room temperature on a shaker, washed with 0.3% PBTriton-X100 2×15 minutes, followed by 2×10 minutes washes with 1× PBS and mounted in Vectashield (Vector Laboratories, CA). A similar protocol was followed for staining stretched cells. The cells were imaged with an upright Leica SPE confocal microscope with a 40× water immersion objective.

### Morphometric analysis

A quantitative cell morphology analysis was performed for each experimental condition (50–100 cells) using Fiji (http://fiji.sc/). Each microscope image was transformed to 8 bits, the scale removed so all the values were in pixels, a threshold set to determine cell borders and the gaps were binary-filled.

Images were analyzed using measurement plugins. The morphological parameters evaluated were: Area - Area of selection in square pixels; Perimeter - The length of the outside boundary of the selection; Circularity - 4π * area/perimeter^2^. A value of 1.0 indicates a perfect circle. As the value approaches 0.0, it indicates an increasingly elongated shape, AR (Aspect Ratio) - major_axis/minor_axis; Roundness - 4 * area/(π * major_axis^2^); Solidity - area/convex area; and Perimeter/Area (or complexity), which is indicative of the complexity of protrusive elements. Statistical analyses were performed with Microsoft Excel. For each type of measurement, the samples were compared 2×2 using parametric t-tests. P values are presented in Table S1.

### Cell stretching: Stage Flexer

The custom-built Stage Flexer consists of a double ringed frame, with an inverted cup-like plastic structure that supports a matrix bonded silicone rubber membrane in a single 35 mm well (Figure S7). The culture membrane is fixed in position above the plastic support with the help of a rubber seal through a groove in between the two rings. The membrane can be deformed by suction from below by applying negative vacuum pressure through an inlet, which induces a uniform deformation in all directions causing the membrane to stretch. The vacuum is applied through an inlet drilled in the lower ring. The amount of strain deformation applied to the flexible substrate was calibrated as described [49], and in our experiments was routinely set at 2.5% uniform stretch. Cells growing on the deformable membrane are accordingly subjected to mechanical stress by centrifugal stretch. The silicone deformable membranes were coated with collagen. For experiments with living cells the Stage Flexer set up was assembled into a sealed humid chamber.

### FLIM experiments

Frequency-domain FLIM experiments on transiently transfected *Drosophila* cells were performed using a Nikon TE2000-U inverted wide-field microscope and a Lambert Instruments Fluorescence Attachment (LIFA; Lambert Instruments, Roden, The Netherlands) for lifetime imaging [50]. A light-emitting diode (Lumiled LUXEON III, λ max = 443 nm) modulated at 40 MHz was used to excite mCFP. Fluorescence detection was performed by a combination of a modulated (40 MHz) image intensifier (II18MD; Lambert Instruments) and a CCD camera (CCD-1300QD; VDS Vosskühler, Osnabrück, Germany) used at 2×2 binning (640×512 pixels). The emission of mCFP was detected through a narrow emission filter (475/20 nm; Semrock, Rochester, U.S.A.) to suppress any crosstalk from mYFP fluorescence emission. FLIM measurements were calibrated with a 50 µM solution of pyranine (HPTS), the lifetime of which was set to 5.4 ns [51]. All FLIM images were calculated from phase stacks of 12 recorded images, with exposure times of individual images of *Drosophila* cells ranging from 200 to 400 ms. Fluorescence lifetimes were calculated for regions of interest (ROIs) comprising individual cells. A number of ∼75 cells (ROIs) were selected for each condition. The obtained FL (pixel) values determined for each individual cell were summed to get FL histograms. These were fitted to Gaussian functions by using the OriginLab 6.0 software, from which the centers of the distributions and the distribution widths were extracted (The FL errors reported (±) are ½ the distribution width of the FL histograms). The experiments were performed at least three times and the data integrated into the histograms. Acceptor photobleaching was performed using an USH-102DH 100 W mercury lamp (Nikon-narrow excitation filter 530/40 nm).

### RNAi treatment

S2R+ cells were co-transfected with specific dsRNAs together with the pAc-dJun-FRET biosensor using approximately 5 µg of dsRNA for each reaction. Talin (DRSC11300) and Myospheroid (DRSC29061) dsRNAs were obtained from the Drosophila RNAi Screening Center (DRSC). These dsRNAs of 507 bp (talin) and 326 bp (Myospheroid) have no off-targets in the *Drosophila* genome (http://fly.rnai.org). On the 4^th^ day after transfection, the cells were replated on collagen or Con-A-coated silicone membranes and subjected to vacuum-assisted stretch FLIM analysis the following day.

To analyze the morphology of the dsRNA treated cells, they were fixed in situ and stained with phalloidin-TRITC before or after 1 hour of mechanical stretch and imaged with a Leica SPE confocal microscope.

## Supporting Information

Figure S1 **Morphometric analysis and FRET-FLIM readouts for S2R+ cells treated with different compounds.** Averaged Area, Perimeter, Perimeter/Area Ratio, Circularity, Aspect Ratio, Roundness and Solidity of S2R+ cells plated on plastic (P) or collagen-coated silicone membranes (C-S) treated with LPS, EGF or L-JNKI1 (JNKI) were calculated for each condition from individual measurements of 50–100 individual cells (see Materials and Methods). Error bars represent Standard Deviations. FRET-FLIM values for the dJun-FRET biosensor were determined as described (Material and Methods). Both, on plastic and on collagen-coated silicone membranes, treatment with LPS result in a significant reduction of FL of S2R+ cells, which associated to an increase in area and perimeter and a reduction of circularity. Treatment with the JNK inhibitor L-JNKI1 of S2R+ cells plated on collagen-coated silicone membranes enhanced the FL, increased the cells perimeter (without affecting the area) and reduced their circularity dramatically. Exposure to EGF of cells plated on plastic had no effect on JNK activity but resulted in an increase of the cells area and perimeter and in a strong reduction of circularity.
(TIF)

Figure S2 **FRET-FLIM quantification of dJun-FRET biosensor controls.** S2R+ cells were transiently transfected with control mCFP-dJun (A) and mCFP (B) biosensors, and fluorescence lifetimes (FL) of mCFP were collected 48 hours post transfection. Cells were left untreated (black) or subjected to treatment with LPS, a JNK signaling activator (red) or L-JNKI1, a JNK inhibitor (blue) for 2 hours before FLIM measurements. Curves represent FLIM data recorded from ∼75 cells for each condition. The chemical activator and inhibitor had no effect on the donor FL of the control sensors.
(TIF)

Figure S3 **Epistatic inhibition of LPS activation of dJun-FRET by the JNK inhibitor L-JNKI1.** S2R+ cells transiently transfected with dJun-FRET were treated with LPS for 2 hours, then washed and treated with the L-JNKI1 for 5 hours. mCFP donor FL data were collected from resting cells (black), LPS treated cells (red) and LPS/L-JNKI1 treated cells (blue). L-JNKI1 was epistatic and reverted the donor FL in activated cells to resting values.
(TIF)

Figure S4 **Morphometric analysis and FRET-FLIM readouts for S2R+ cells subjected to mechanical stretch.** Averaged Area, Perimeter, Perimeter/Area Ratio, Circularity, Aspect Ratio, Roundness and Solidity of S2R+ cells plated on collagen-coated silicone membranes, untreated (WT) or subjected to RNA interference for β-integrin (Mys-) or talin (Talin-), or plated on concanavalin A-coated silicone membranes (ConA). Morphometric parameters were collected for each condition from individual measurements of 50–100 individual cells (see Materials and Methods) before (Unstretched) or after 2 hours of static vacuum-assisted stretch (Stretched). Error bars represent Standard Deviations. FRET-FLIM values for the dJun-FRET biosensor were determined as described (Material and Methods) for the same conditions. S2R+ cells plated on collagen-coated silicone membranes presented low levels of JNK activity (high FL), which robustly increased upon cell stretching. Stretching also results in a moderate reduction of their areas and circularity and a modest increase of their complexity (Perimeter/Area). Inhibiting β-integrin did not affect S2R+ cells area but elicited an increase in JNK activity and cell complexity and promoted a reduction of circularity and solidity. Upon stretching, the level of JNK activity of these cells was not affected, neither their size, but their complexity increased and their circularity and solidity were further reduced. Same effects in terms of JNK activity were observed for S2R+ cells plated on concanavalin A-coated silicone membranes. The FL of unstretched cells was similar to β-integrin RNAi treated cells and it did not change upon stretching. These cells, however display a very different morphology. They flattened dramatically, showed very low complexity and presented high circularity. None of these parameters were affected by mechanical stretch. Talin inhibition in unstretched conditions resulted in an activation of the JNK pathway higher than that observed for β-integrin inhibition or for cells plated on concanavalin A. The morphology of these cells was somehow reminiscent of that of β-integrin deficient cells, areas were small, complexity elevated (a bit higher) and circularity and solidity low. Stretching, however, lead to a different response. Contrary to the response of β-integrin deficient cells to stretch, the absence of talin did not prevent further activation of the JNK pathway and resulted in the reduction of cell complexity and the enhancement of circularity and solidity upon stretching.
(TIF)

Figure S5 **Time Lapse FLIM of dJun-FRET activation by mechanical stretch.** S2R+ cells transiently transfected with dJun-FRET were plated on collagen-coated silicone membranes mounted in the Stage Flexer setup. mCFP donor FL was collected at intervals of 5 minutes for 30 minutes without stretch. After 30 minutes, mechanical stretching was applied and FLs were further recorded at 5 minutes intervals for 4 hours. Average and standard deviations for FL values from 10 different measurements (ROIs) are plotted. Red arrows indicate the time point at which vacuum was switched on. A substantial decrease in FL could be observed within 20 minutes of stretching and a plateau is reached in less than 2 hours. JNK activation remains stable from this time point onwards.
(TIF)

Figure S6 **Morphometric analysis and FRET-FLIM readouts for S2R+ cells plated on different substrates.** Averaged Area, Perimeter, Perimeter/Area Ratio, Circularity, Aspect Ratio, Roundness and Solidity of S2R+ cells plated on plastic, glass, collagen-coated silicone membranes [Col (S)], collagen-coated glass [Col (G)], concanavalin A-coated silicone membranes [ConA (S)] and concanavalin A-coated glass [ConA (G)] were calculated for each condition from individual measurements of 50–100 individual cells (see Materials and Methods). Error bars represent Standard Deviations. FRET-FLIM values for the dJun-FRET biosensor were determined as described (Material and Methods). The choice of substrate affects the level of JNK activity and the morphology of S2R+ cells. On plastic, S2R+ cells grew small and present high FL values. On glass, however, cells are bigger and show high JNK activity (low FL). Both conditions resulted in moderate complexity (Perimeter/Area) and Circularity. Alternatively, plating cells on collagen lead to small sizes and elicited low levels of JNK activation (somehow enhanced on collagen-coated glass, which might be due to the rigidity of the surface). These cells are relatively more complex and tend to show smaller circularity. Finally, plating cells on concanavalin A-coated surfaces steered cell flattening, low complexity and high circularity and intermediate levels of JNK activity.
(TIF)

Figure S7 **Mechanical Stretch Device.** A custom-built Stage Flexer set up was used to induce mechanical stress. The Stage Flexer consists of a double ringed frame with an inverted cup-like plastic structure that supports a matrix bonded silicone rubber membrane in a single 35 mm well. The membrane is fixed in position above the plastic support with the help of a rubber seal. The vacuum is applied through an inlet drilled in the lower ring. The source of vacuum when turned on sucks the membrane from below, inducing a uniform deformation of the membrane in all directions, causing its stretching.
(TIF)

Table S1 **Statistical significance of morphometric quantification comparisons.** Parametric t-tests P values for the individual morphometric comparisons described in the text are displayed in a two entries table. In red are shown those comparisons with differences statistically significant at P values<0.001, in dark brown, those comparisons with differences statistically significant at P values<0.005 and in black, those comparisons with no significant differences.
(PDF)

Text S1 **Integrin-dependent activation of the JNK signaling pathway by mechanical stress.**
(DOC)

Movie S1 **Dynamics of S2R+ cells response to mechanical stretch.** S2R+ cells transiently transfected with pMT-GFP-Tubulin were plated on collagen-coated silicone membranes and mounted in a Stage Flexer set up. We performed time-lapse imaging of the cells using an upright Leica SPE confocal microscope with a 40× water immersion objective at intervals of 3 minutes. Recording lasted for 45 minutes at resting condition and for a further 60 minutes upon induction of static stretch. Before stretch, S2R+ cells display active cytoskeletal dynamics and stably maintain a stretched morphology. In response to mechanical stress, plated cells withdraw protrusions and retract, rounding up by 60 minutes. In this period, the donor FL shifts (black to red) from 2.43±0.15 ns to 2.18±0.15 ns reflecting an increase in JNK signaling.
(MOV)
