# Supplementary figures and images for: Integrin-Dependent Activation of the JNK Signaling Pathway by Mechanical Stress
Source: PLoS One. 2011 Dec 13;6(12):e26182. doi: 10.1371/journal.pone.0026182 (PMC3236745; doi:10.1371/journal.pone.0026182)

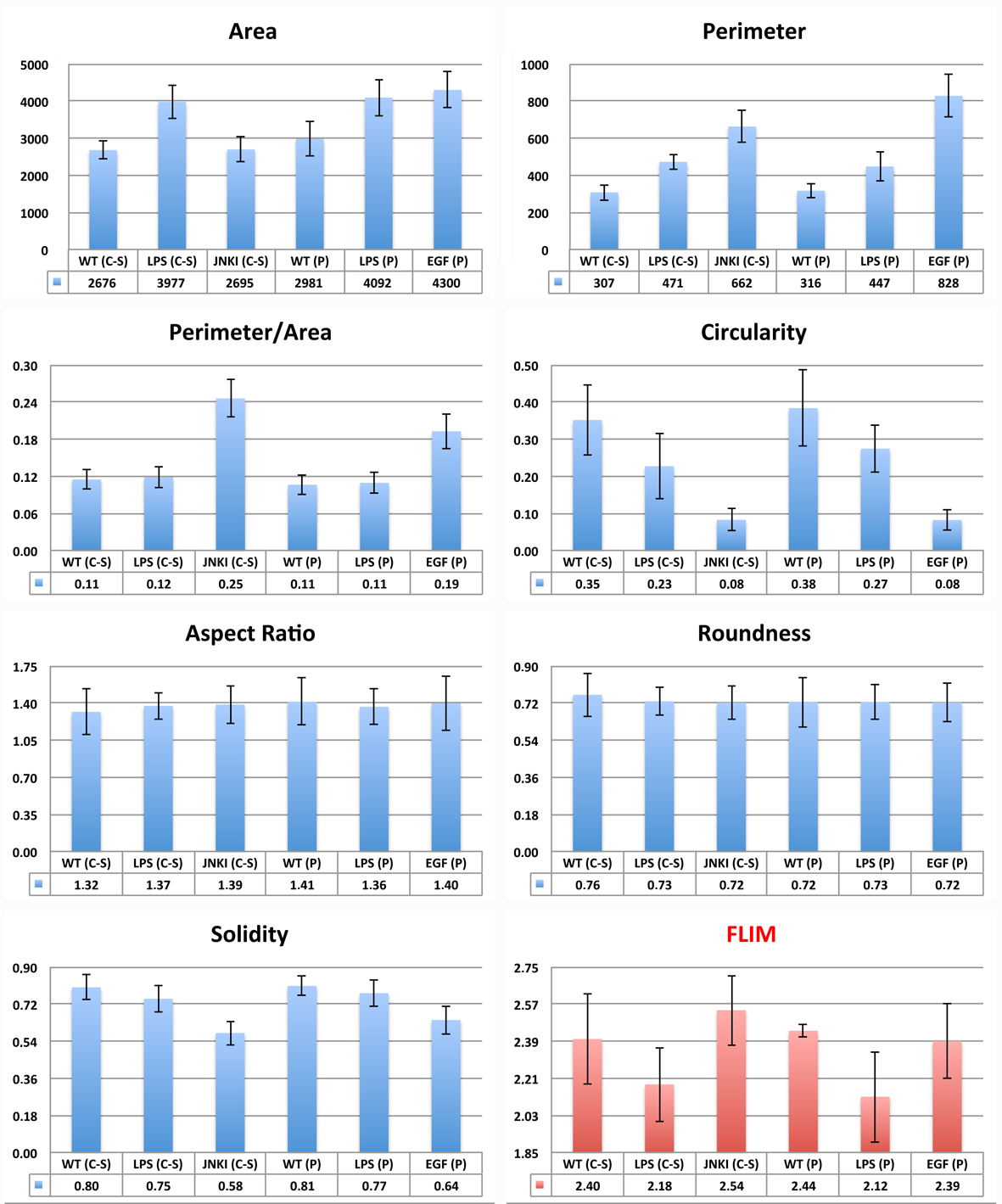

Supplement: Figure S1 — Morphometric analysis and FRET-FLIM readouts for S2R+ cells treated with different compounds. Averaged Area, Perimeter, Perimeter/Area Ratio, Circularity, Aspect Ratio, Roundness and Solidity of S2R+ cells plated on plastic (P) or collagen-coated silicone membranes (C-S) treated with LPS, EGF or L-JNKI1 (JNKI) were calculated for each condition from individual measurements of 50–100 individual cells (see Materials and Methods). Error bars represent Standard Deviations. FRET-FLIM values for the dJun-FRET biosensor were determined as described (Material and Methods). Both, on plastic and on collagen-coated silicone membranes, treatment with LPS result in a significant reduction of FL of S2R+ cells, which associated to an increase in area and perimeter and a reduction of circularity. Treatment with the JNK inhibitor L-JNKI1 of S2R+ cells plated on collagen-coated silicone membranes enhanced the FL, increased the cells perimeter (without affecting the area) and reduced their circularity dramatically. Exposure to EGF of cells plated on plastic had no effect on JNK activity but resulted in an increase of the cells area and perimeter and in a strong reduction of circularity. (TIF) [file pone.0026182.s001.tif]

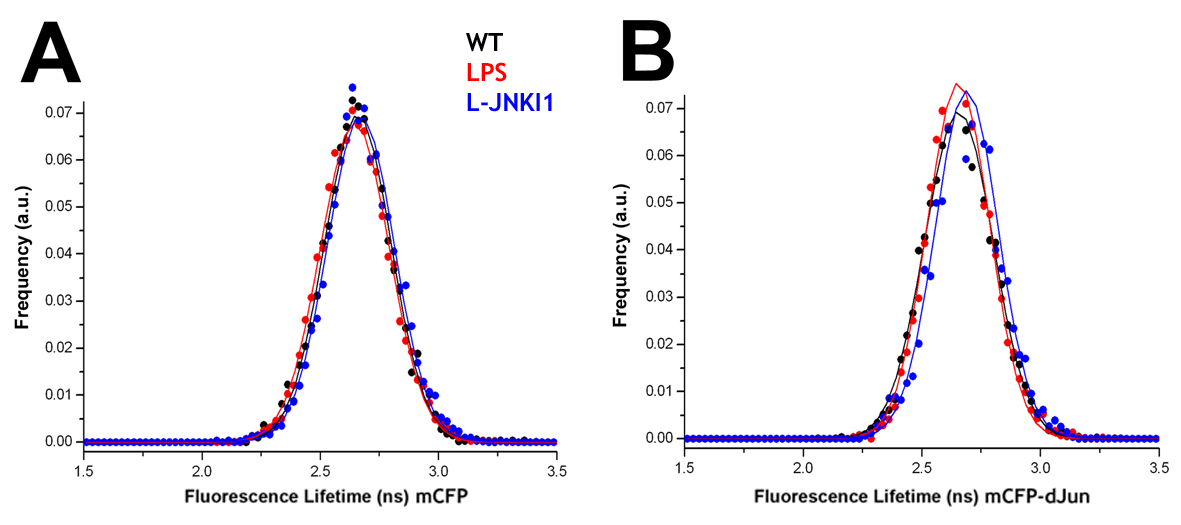

Supplement: Figure S2 — FRET-FLIM quantification of dJun-FRET biosensor controls. S2R+ cells were transiently transfected with control mCFP-dJun (A) and mCFP (B) biosensors, and fluorescence lifetimes (FL) of mCFP were collected 48 hours post transfection. Cells were left untreated (black) or subjected to treatment with LPS, a JNK signaling activator (red) or L-JNKI1, a JNK inhibitor (blue) for 2 hours before FLIM measurements. Curves represent FLIM data recorded from ∼75 cells for each condition. The chemical activator and inhibitor had no effect on the donor FL of the control sensors. (TIF) [file pone.0026182.s002.tif]

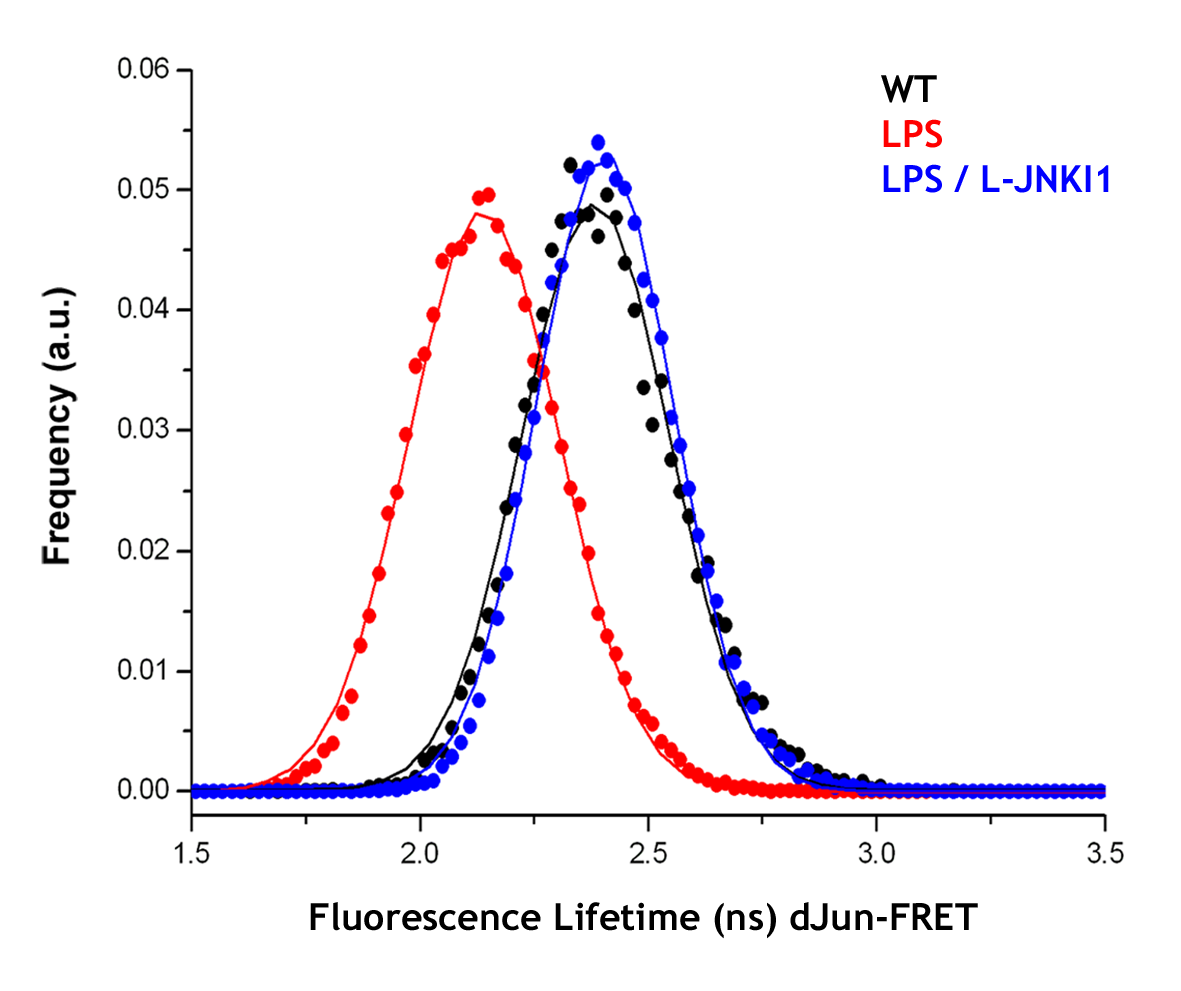

Supplement: Figure S3 — Epistatic inhibition of LPS activation of dJun-FRET by the JNK inhibitor L-JNKI1. S2R+ cells transiently transfected with dJun-FRET were treated with LPS for 2 hours, then washed and treated with the L-JNKI1 for 5 hours. mCFP donor FL data were collected from resting cells (black), LPS treated cells (red) and LPS/L-JNKI1 treated cells (blue). L-JNKI1 was epistatic and reverted the donor FL in activated cells to resting values. (TIF) [file pone.0026182.s003.tif]

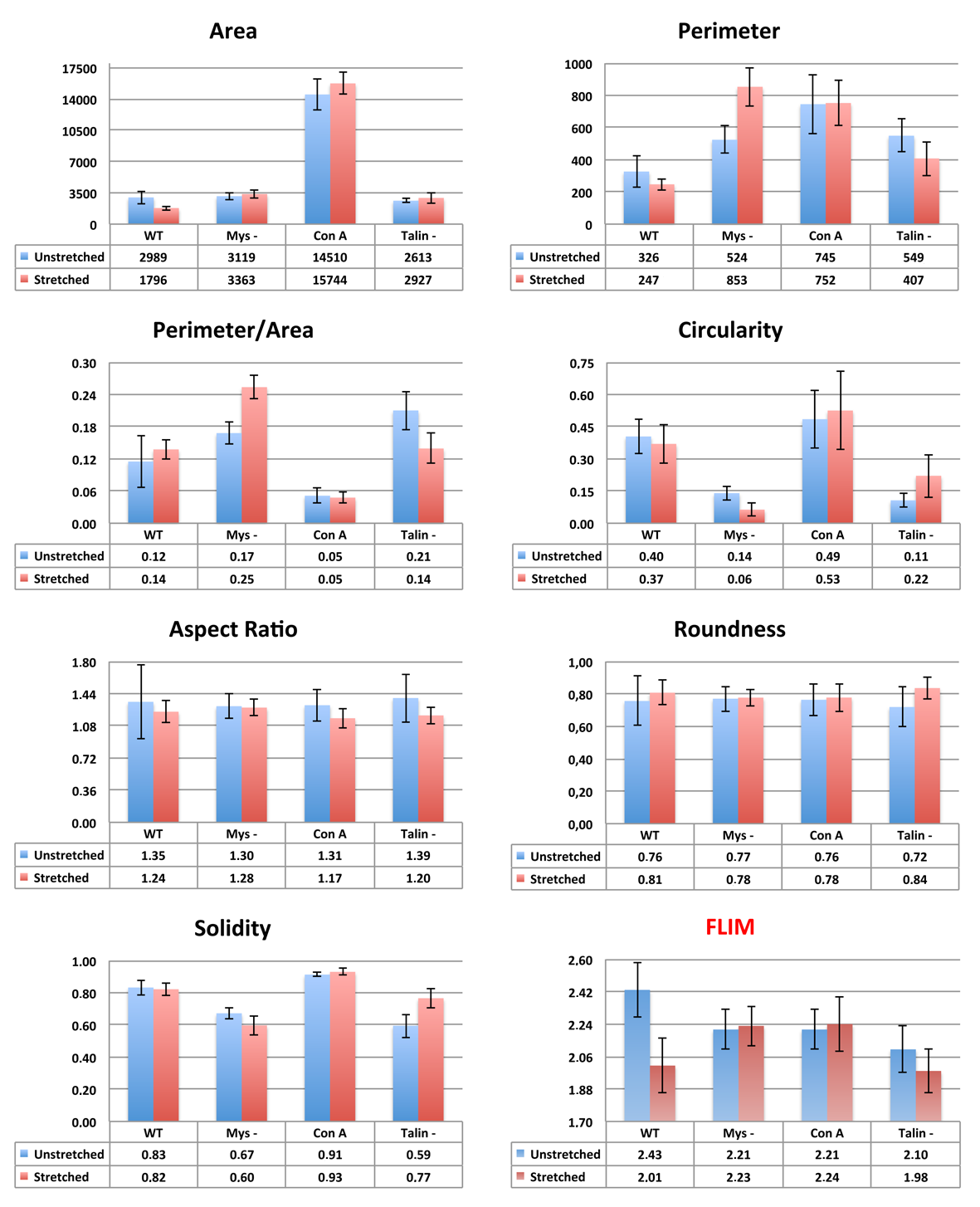

Supplement: Figure S4 — Morphometric analysis and FRET-FLIM readouts for S2R+ cells subjected to mechanical stretch. Averaged Area, Perimeter, Perimeter/Area Ratio, Circularity, Aspect Ratio, Roundness and Solidity of S2R+ cells plated on collagen-coated silicone membranes, untreated (WT) or subjected to RNA interference for β-integrin (Mys-) or talin (Talin-), or plated on concanavalin A-coated silicone membranes (ConA). Morphometric parameters were collected for each condition from individual measurements of 50–100 individual cells (see Materials and Methods) before (Unstretched) or after 2 hours of static vacuum-assisted stretch (Stretched). Error bars represent Standard Deviations. FRET-FLIM values for the dJun-FRET biosensor were determined as described (Material and Methods) for the same conditions. S2R+ cells plated on collagen-coated silicone membranes presented low levels of JNK activity (high FL), which robustly increased upon cell stretching. Stretching also results in a moderate reduction of their areas and circularity and a modest increase of their complexity (Perimeter/Area). Inhibiting β-integrin did not affect S2R+ cells area but elicited an increase in JNK activity and cell complexity and promoted a reduction of circularity and solidity. Upon stretching, the level of JNK activity of these cells was not affected, neither their size, but their complexity increased and their circularity and solidity were further reduced. Same effects in terms of JNK activity were observed for S2R+ cells plated on concanavalin A-coated silicone membranes. The FL of unstretched cells was similar to β-integrin RNAi treated cells and it did not change upon stretching. These cells, however display a very different morphology. They flattened dramatically, showed very low complexity and presented high circularity. None of these parameters were affected by mechanical stretch. Talin inhibition in unstretched conditions resulted in an activation of the JNK pathway higher than that observed for β-integr [file pone.0026182.s004.tif]

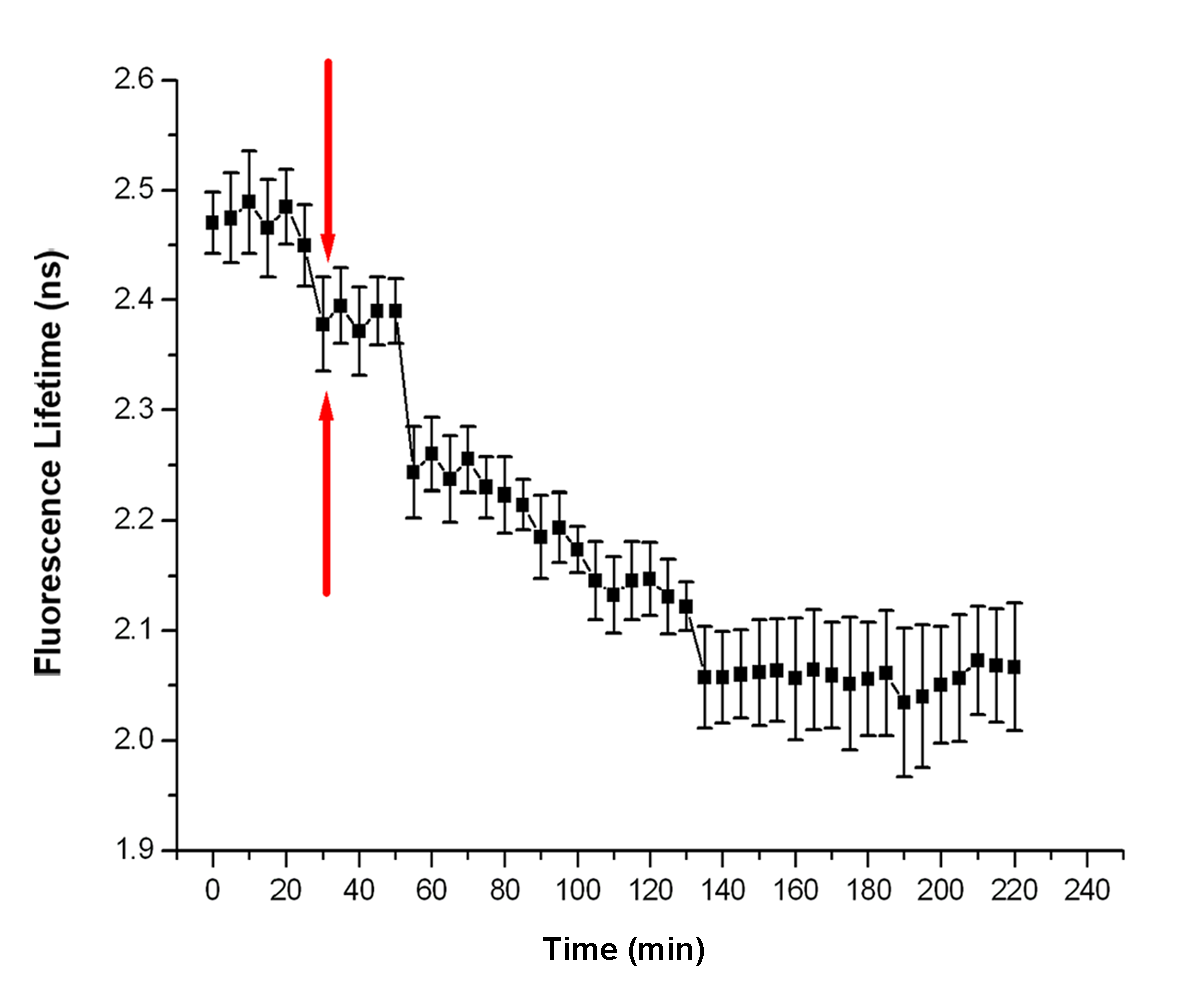

Supplement: Figure S5 — Time Lapse FLIM of dJun-FRET activation by mechanical stretch. S2R+ cells transiently transfected with dJun-FRET were plated on collagen-coated silicone membranes mounted in the Stage Flexer setup. mCFP donor FL was collected at intervals of 5 minutes for 30 minutes without stretch. After 30 minutes, mechanical stretching was applied and FLs were further recorded at 5 minutes intervals for 4 hours. Average and standard deviations for FL values from 10 different measurements (ROIs) are plotted. Red arrows indicate the time point at which vacuum was switched on. A substantial decrease in FL could be observed within 20 minutes of stretching and a plateau is reached in less than 2 hours. JNK activation remains stable from this time point onwards. (TIF) [file pone.0026182.s005.tif]

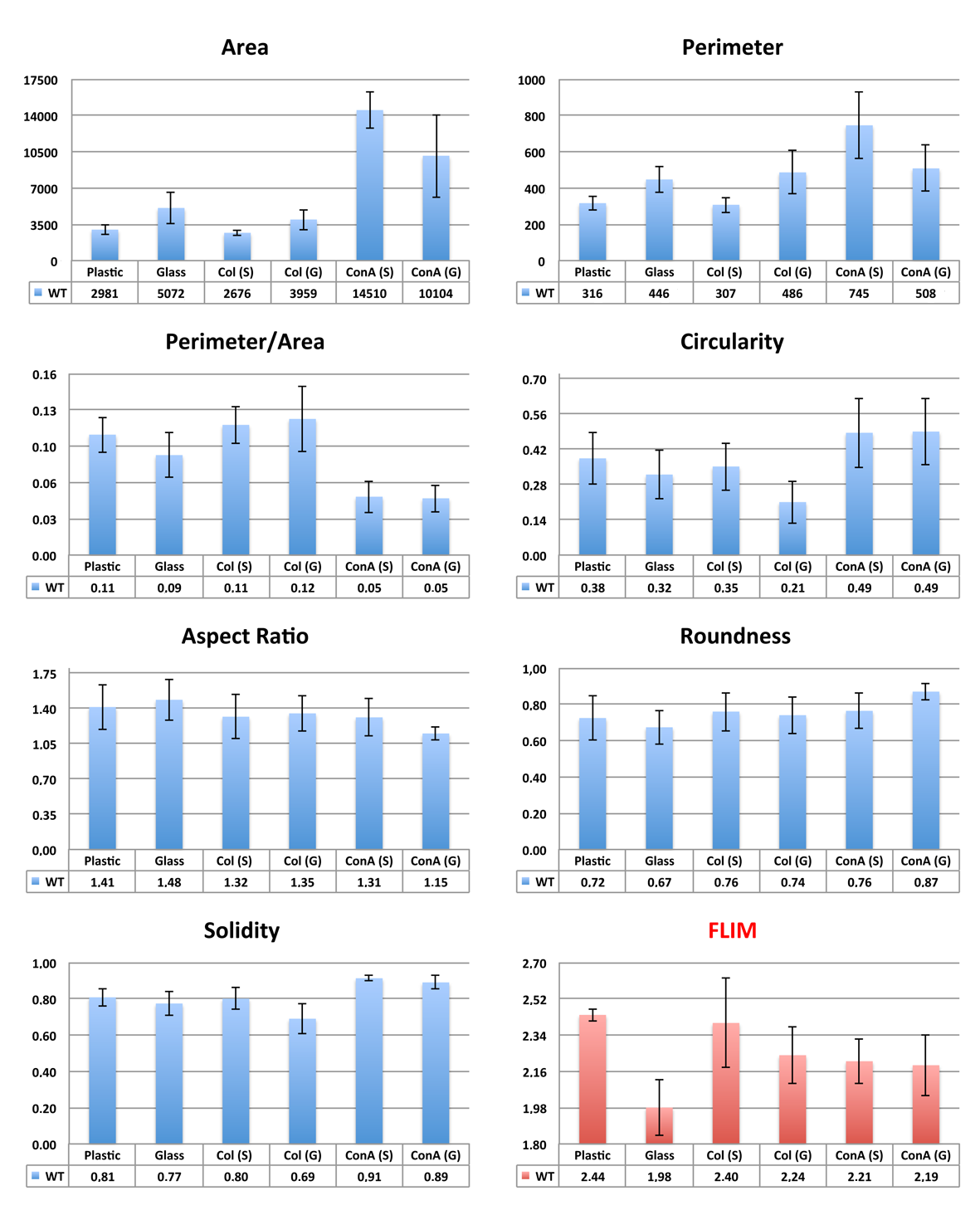

Supplement: Figure S6 — Morphometric analysis and FRET-FLIM readouts for S2R+ cells plated on different substrates. Averaged Area, Perimeter, Perimeter/Area Ratio, Circularity, Aspect Ratio, Roundness and Solidity of S2R+ cells plated on plastic, glass, collagen-coated silicone membranes [Col (S)], collagen-coated glass [Col (G)], concanavalin A-coated silicone membranes [ConA (S)] and concanavalin A-coated glass [ConA (G)] were calculated for each condition from individual measurements of 50–100 individual cells (see Materials and Methods). Error bars represent Standard Deviations. FRET-FLIM values for the dJun-FRET biosensor were determined as described (Material and Methods). The choice of substrate affects the level of JNK activity and the morphology of S2R+ cells. On plastic, S2R+ cells grew small and present high FL values. On glass, however, cells are bigger and show high JNK activity (low FL). Both conditions resulted in moderate complexity (Perimeter/Area) and Circularity. Alternatively, plating cells on collagen lead to small sizes and elicited low levels of JNK activation (somehow enhanced on collagen-coated glass, which might be due to the rigidity of the surface). These cells are relatively more complex and tend to show smaller circularity. Finally, plating cells on concanavalin A-coated surfaces steered cell flattening, low complexity and high circularity and intermediate levels of JNK activity. (TIF) [file pone.0026182.s006.tif]

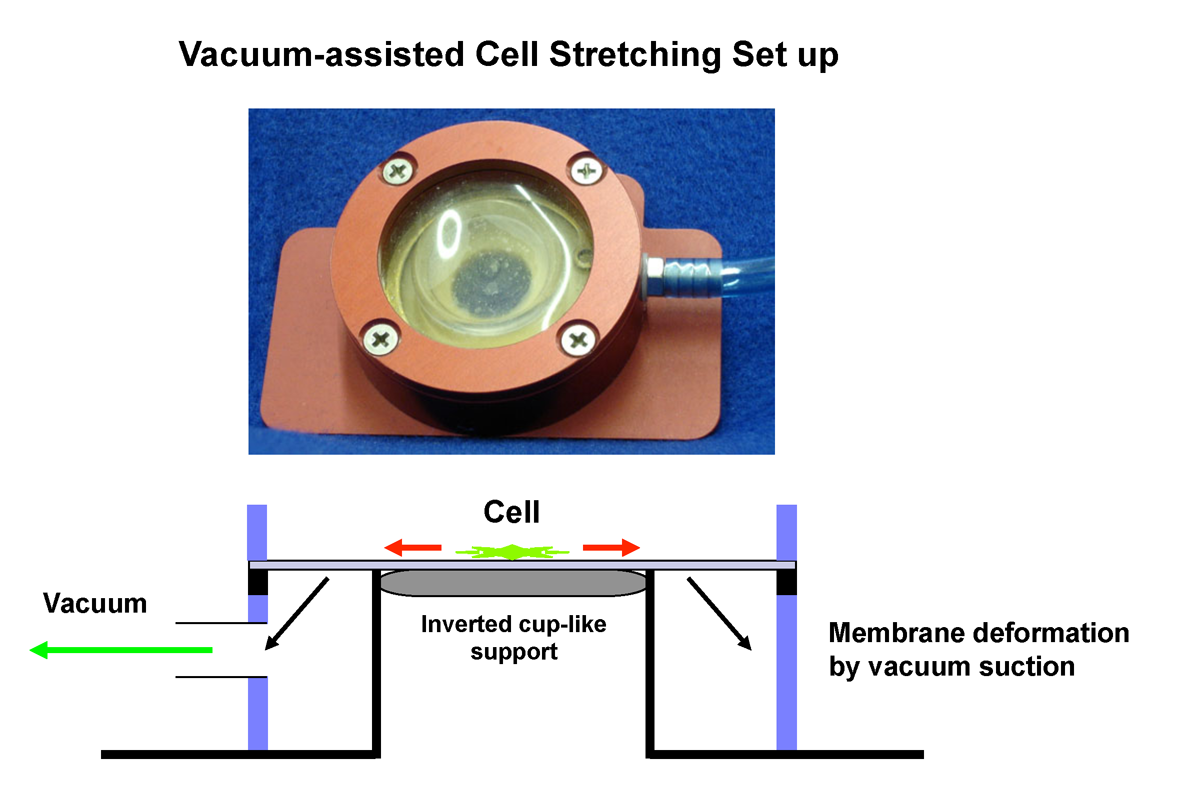

Supplement: Figure S7 — Mechanical Stretch Device. A custom-built Stage Flexer set up was used to induce mechanical stress. The Stage Flexer consists of a double ringed frame with an inverted cup-like plastic structure that supports a matrix bonded silicone rubber membrane in a single 35 mm well. The membrane is fixed in position above the plastic support with the help of a rubber seal. The vacuum is applied through an inlet drilled in the lower ring. The source of vacuum when turned on sucks the membrane from below, inducing a uniform deformation of the membrane in all directions, causing its stretching. (TIF) [file pone.0026182.s007.tif]
