# Supplementary material for: Integrin-Dependent Activation of the JNK Signaling Pathway by Mechanical Stress
Source: PLoS One. 2011 Dec 13;6(12):e26182. doi: 10.1371/journal.pone.0026182 (PMC3236745; doi:10.1371/journal.pone.0026182)
Supplement: Table S1 — Statistical significance of morphometric quantification comparisons. Parametric t-tests P values for the individual morphometric comparisons described in the text are displayed in a two entries table. In red are shown those comparisons with differences statistically significant at P values<0.001, in dark brown, those comparisons with differences statistically significant at P values<0.005 and in black, those comparisons with no significant differences. (PDF) [file pone.0026182.s008.pdf]

Table S1. Statistical significance of morphometric quantification comparisons

	AREA	PERIMETER	PERIMETER/AREA	CIRCULARITY	ASPECT RATIO	ROUNDNESS	SOLIDITY
**FIGURE S1**							
**WT (C-S)/LPS (C-S)**	0,0007	<0.0001	0,53	0,022	0,66	0,4	0,1
**WT (C-S)/L-JNKI1 (C-S)**	0,74	<0.0001	<0.0001	<0.0001	0,3	0,21	<0.0001
**LPS (C-S)/L-JNKI1 (C-S)**	<0.0001	<0.0001	<0.0001	<0.0001	0,51	0,68	<0.0001
**WT (P)/LPS (P)**	<0.0001	<0.0001	0,88	0,0013	0,44	0,65	0,2
**WT (P)/EGF (P)**	<0.0001	<0.0001	<0.0001	<0.0001	0,71	0,76	<0.0001
**LPS (P)/EGF (P)**	0,38	<0.0001	<0.0001	<0.0001	0,79	0,94	<0.0001
**WT (C-S)/WT (P)**	0,64	0,69	0,98	0,71	0,22	0,59	0,73
**FIGURE S4**							
**Unstretched**							
**WT/Mys-**	0,59	<0.0001	0,0005	<0.0001	0,74	0,63	<0.0001
**WT/ConA**	<0.0001	<0.0001	0,0002	0,03	0,88	0,75	<0.0001
**WT/Talin-**	0,13	<0.0001	<0.0001	<0.0001	0,53	0,63	<0.0001
**Stretched**							
**WT (S)/Mys- (S)**	<0.0001	<0.0001	<0.0001	<0.0001	0,38	0,28	<0.0001
**WT (S)/Con-A (S)**	<0.0001	<0.0001	<0.0001	0,0031	0,3	0,43	<0.0001
**WT (S) /Talin- (S)**	<0.0001	<0.0001	0,87	0,0098	0,38	0,42	0,029
**Unstretched/Stretched**							
**WT**	<0.0001	0,0089	0,044	0,37	0,27	0,14	0,45
**Mys-**	0,16	<0.0001	<0.0001	0,0002	0,78	0,94	0,0034
**ConA**	0,19	0,93	0,44	0,56	0,12	0,82	0,073
**Talin-**	0,11	0,0036	<0.0001	0,0004	0,0079	0,0068	<0.0001
**FIGURE S6**							
**WT (G)/Collagen (G)**	0,009	0,25	0,0091	0,0058	0,49	0,34	0,018
**WT (G)/Con-A (G)**	0,0005	0,094	<0.0001	0,0018	<0.0001	<0.0001	<0.0001
**Collagen (G)/Con-A (G)**	<0.0001	0,3	<0.0001	<0.0001	0,0004	<0.0001	<0.0001
**WT (G)/WT (P)**	<0.0001	<0.0001	0,035	0,25	0,78	0,22	0,4
**Collagen (S)/Collagen (G)**	0,0054	<0.0001	0,23	<0.0001	0,49	0,56	<0.0001
**Con-A (S)/Con-A (G)**	0,13	0,011	0,52	0,92	0,0028	0,0016	0,13
