# Supplementary material for: Integrin-Dependent Activation of the JNK Signaling Pathway by Mechanical Stress
Source: PLoS One. 2011 Dec 13;6(12):e26182. doi: 10.1371/journal.pone.0026182 (PMC3236745; doi:10.1371/journal.pone.0026182)
Supplement: Text S1 — Integrin-dependent activation of the JNK signaling pathway by mechanical stress. (DOC) [file pone.0026182.s009.doc]

Integrin-Dependent Activation of the JNK Signaling Pathway by Mechanical Stress

Andrea Maria Pereira, Cicerone Tudor, Johannes S. Kanger, Vinod Subramaniam and Enrique Martin-Blanco

**SUPPLEMENTAL INFORMATION**

**FRET and FLIM**

Recent advances in fluorescence microscopy techniques combined with the development of genetically encoded modified fluorescent proteins (FPs) have allowed direct monitoring of protein dynamics and interactions. Among the range of available techniques, the combination of FRET (Förster Resonance Energy Transfer) and FLIM (Fluorescence Lifetime Imaging Microscopy) is ideal for the study of protein-protein interactions in living cells or for the analysis of protein conformational changes . For FRET, two suitable fluorophores (‘donors’ and ‘acceptors’) are associated with proteins of interest. If the two proteins come into close proximity, then the energy from the donor is non-radiatively transferred to the acceptor . Both *in vitro* and within cells, the fluorescence lifetime (FL) of a fluorophore is sensitive to changes in the local environment of the fluorophore, such as changes in pH or ion concentration, and is also influenced by energy transfer to other fluorophores in close proximity. When FRET occurs, an additional decay channel is available to the excited state of the donor fluorophore, resulting in the decrease of the FL of the donor molecule. Thus, the occurrence of FRET can be measured by monitoring the change in donor FL in the presence and absence of a suitable acceptor. FLIM determination of the activity of FRET biosensors in living cells is a powerful tool for characterizing in real time the activity of signaling transduction networks and the study of their role in the control of cellular function in response to extrinsic or intrinsic physico-chemical stimuli.

**SUPPLEMENTAL REFERENCES**

1. Lippincott-Schwartz J, Altan-Bonnet N, Patterson GH (2003) Photobleaching and photoactivation: following protein dynamics in living cells. Nat Cell Biol Suppl: S7-14.

2. Lleres D, Swift S, Lamond AI (2007) Detecting protein-protein interactions in vivo with FRET using multiphoton fluorescence lifetime imaging microscopy (FLIM). Curr Protoc Cytom Chapter 12: Unit12 10.

3. Calleja V, Ameer-Beg SM, Vojnovic B, Woscholski R, Downward J, et al. (2003) Monitoring conformational changes of proteins in cells by fluorescence lifetime imaging microscopy. Biochem J 372: 33-40.

4. Elangovan M, Wallrabe H, Chen Y, Day RN, Barroso M, et al. (2003) Characterization of one- and two-photon excitation fluorescence resonance energy transfer microscopy. Methods 29: 58-73.
